# Core Eigenmodes and their Impact on the Earth’s Rotation

**DOI:** 10.1007/s10712-021-09668-y

**Published:** 2021-11-10

**Authors:** Santiago A. Triana, Mathieu Dumberry, David Cébron, Jérémie Vidal, Antony Trinh, Felix Gerick, Jérémy Rekier

**Affiliations:** 1 Royal Observatory of Belgium, Ringlaan 3, BE-1180 Brussels, Belgium; 2 Department of Physics, University of Alberta, Edmonton, AB T6G 2E1 Canada; 3 ISTerre CS 40700, Université Grenoble Alpes, 38058 Cedex 9 Grenoble, France; 4 Lunar and Planetary Laboratory, University of Arizona, 1629 E. University Blvd, P.O. Box 210092, Tucson, AZ 85721-0092 USA

**Keywords:** Core modes, Rotational modes, Earth rotation

## Abstract

Changes in the Earth’s rotation are deeply connected to fluid dynamical processes in the outer core. This connection can be explored by studying the associated Earth eigenmodes with periods ranging from nearly diurnal to multi-decadal. It is essential to understand how the rotational and fluid core eigenmodes mutually interact, as well as their dependence on a host of diverse factors, such as magnetic effects, density stratification, fluid instabilities or turbulence. It is feasible to build detailed models including many of these features, and doing so will in turn allow us to extract more (indirect) information about the Earth’s interior. In this article, we present a review of some of the current models, the numerical techniques, their advantages and limitations and the challenges on the road ahead.

## **Article Highlights**

- Fluid motion within the Earth’s core can induce measurable changes in the rotation and magnetic field of the planet
- We review techniques to study these flows as normal modes, together with their interplay with rotational modes
- A proper understanding of mode excitation is essential to interpret observations as well as numerical simulations

## Introduction

The normal modes of oscillation of our planet come in different shapes and kinds. Beyond the well-known seismic modes that have helped immensely to obtain information about the Earth’s interior, we have periodic variations in the Earth’s global rotation as well as oscillations in the fluid outer core supported by the Coriolis force. While seismic modes have periods shorter than one hour, rotational and Coriolis-supported fluid core oscillations have periods typically longer than 12 hours (as measured in a reference frame attached to the rotating mantle). The translational modes of the inner core, i.e., the Slichter modes, not discussed in this review, have a period in between, approximately six hours. Just like the seismic modes, the observation of rotational and fluid core modes can also help us improve our knowledge of the Earth’s interior. Models to study the rotational variations such as nutations are sophisticated enough to include viscoelastic deformations of the mantle and solid inner core, but only include limited aspects of the dynamics in the fluid core. Conversely, studies focused on dynamics in the fluid core often assume completely rigid fluid-solid boundaries, together with *prescribed* motions of the solid regions. Thus, an interdisciplinary approach is required if we are to understand the interplay between rotational variations and fluid core dynamics and use it to better constrain the Earth’s internal structure, dynamics and evolution. We present the material in this article with that spirit in mind.

The extreme values of some of the physical parameters in the Earth’s core pose an enormous challenge for numerical studies of core eigenmodes. There is also an observational challenge since, as we discuss briefly in this article, direct detection of these modes is very unlikely. One might wonder then about the usefulness of the study of such modes. The answer is that the signature of many of these modes might still be present in Earth’s nutation or magnetic measurements, but clearly we would not know what to look for if we do not have a clear picture of their properties at hand. Conversely, if we manage to detect their presence, knowledge of their physical characteristics would immediately give us valuable insight into the structure of the core. Yet another reason is that having a sound physical picture of the modes is very helpful to clarify and disentangle results from other studies, experimental or numerical (direct numerical simulation, DNS, for instance), that cannot reach extreme geophysical parameters either.

Typically, studies on the Earth’s rotational variations employ quantities with physical dimensions, which are convenient when comparing theory against geodetic measurements. In contrast, fluid core dynamics studies use *dimensionless* quantities as they are more suited for numerical computations. We follow this convention in this review, using both dimensional and dimensionless quantities depending on the topic at hand.

The undulatory behavior of the core flow is sometimes referred to in the literature as a ‘wave’ (propagating as in unbounded media, notably during transient stages) or as a ‘mode’ in bounded geometries (i.e., when the boundary conditions play a central role, often on longer timescales). We follow loosely this convention. Note, however, that there is no standard convention in the literature. Some authors refer to bounded, non-axisymmetric inertial eigenmodes as inertial ‘waves’, reflecting the fact that these modes drift in the azimuthal direction, while using the term ‘oscillations’ only for the axisymmetric modes (Zhang et al. 2004; Greenspan 1968).

We begin by presenting some of the techniques used to model the eigenmodes associated with the fluid outer core in Sect. 2 and give a physically motivated discussion of how these modes are affected by magnetic fields, density stratification or the presence of the inner core in Sect. 3. We introduce the global rotational modes of the Earth using the angular momentum approach in Sect. 4. We proceed to describe a simple but fully coupled model whose eigenmodes include both rotational and inertial modes in Sect. 5. We follow with a discussion on the geophysical applications and current challenges in Sect. 6. The conclusions and future outlook section closes this paper in Sect. 7.

## Modeling of the Dynamics of the Earth’s Outer Core

### Idealized Model

The full set of equations governing the dynamics of the Earth’s liquid core describes the time evolution of the velocity, density, energy and the magnetic field. These equations are a challenge to solve analytically or numerically and, thus, idealizations and approximations are often used. We present in this section the ingredients that idealized Earth models typically include to study its dynamics.

**Fig. 1:**
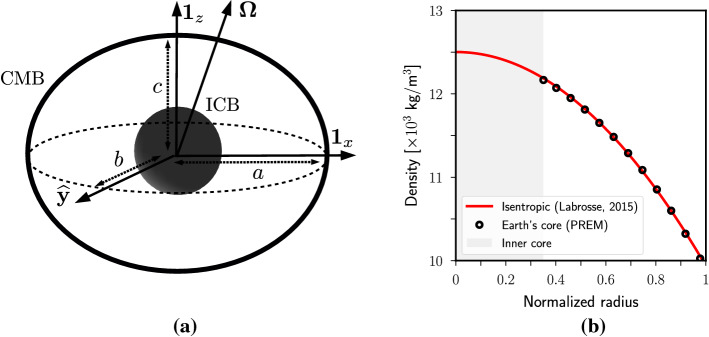
(a) Geometry of the Earth’s core model. The CMB has been represented by a triaxial ellipsoid of semi-axes [*a*, *b*, *c*]. (b) Density in the Earth’s core as a function of the radius (normalized by equatorial radius $R_{o}=3480$ km of the CMB). Open circles: PREM values (Dziewonski and Anderson 1981). Red curve: isentropic model (Labrosse 2015). Gray area illustrates the inner core

The Earth’s liquid core is modeled as a solid shell of volume ${\mathcal {V}}$ filled with an electrically conducting Newtonian fluid of density $\rho _f$, uniform kinematic viscosity $\nu _f$, electrical conductivity $\gamma$ and magnetic diffusivity $\eta =1/(\gamma \mu )$ (with $\mu$ the magnetic permeability). The fluid is enclosed by a solid mantle and has a solid inner core in its center, as illustrated in Fig. 1(a). The core–mantle boundary (CMB) is not perfectly spherical, with global (polar and equatorial) elliptical deformations on which smaller wavelengths are superimposed (see figure 6 in Koelemeijer 2020). The CMB is modeled sometimes in the literature as a triaxial ellipsoid, although oblate spheroidal or spherical approximations are commonly used. Although there is evidence of non-hydrostatic effects (Wu and Wahr 1997) determining the shape of the CMB, the expected hydrostatic flattening ($\sim 1/412$) of the inner-core boundary (ICB) is only slightly smaller than the CMB’s hydrostatic flattening ($\sim 1/392)$. However, for simplicity, most models represent the ICB as a sphere of radius $r_{i}=0.35 \, R_0$ with $R_0$ being the equatorial radius of the CMB.

Models need a reference equilibrium state upon which fluid motions develop. Such a state can be defined by assuming a fluid in hydrostatic equilibrium, stratified in density and rapidly rotating at angular velocity $\varOmega _0$ in the inertial frame. The radial density gradient results from variation of the temperature and of the fraction of light elements in the core (Alfe et al. 2003; Gubbins et al. 2004). The seismically inferred density profile (e.g., as provided by the PREM model, see in Dziewonski and Anderson 1981) only varies by approximately 20% from the ICB to the CMB (see Fig. 1b), so the effects of compressibility are relatively weak. Moreover, the density variations are found to be very close to an adiabatic density profile (Labrosse 2015). Consequently, some models adopt the Boussinesq approximation (Anufriev et al. 2005), which neglects the density variations about the adiabatic profile except in the buoyancy force where they are retained. In this setup, the variations of density are due to variations of temperature and composition of light elements. $[\alpha _T,\alpha _C]$ denote the thermal and compositional expansion coefficients. The temperature and composition fields are also associated with the thermal and compositional diffusion coefficients $[\kappa _T,\kappa _C]$ in the Boussinesq approximation. To work with dimensionless variables, which is customary in fluid dynamics, we also introduce a number of unit scales. We use $\varOmega _0^{-1}$ as the time scale, the radius $R_o$ of the CMB as the length scale, the typical amplitude $B_0$ of the magnetic field at the CMB as the magnetic scale, $\varOmega _0^2 R_o/(\alpha _T g_o)$ as the temperature scale (with $g_o$ the scalar gravitational acceleration at the CMB), and $\varOmega _0^2 R_o/(\alpha _C g_o)$ as the scale for the mass fraction of light elements.

We employ the frame of reference attached to the mantle rotating at the angular velocity ${\varvec{\varOmega }}$, with $[{\varvec{1}}_x, {\varvec{1}}_y, {\varvec{1}}_z]$ denoting the unit Cartesian vectors where ${\varvec{1}}_z$ is chosen as the mean axis of rotation. We write the gravitational field ${\varvec{g}} = - \nabla \varPhi$, and seek small perturbations of the temperature and of the mass fraction of light elements upon the stratified state $[T_0 (\varPhi ), C_0 (\varPhi )]$ that measures the departure of the background stratification from isentropic equilibrium. The stratification of the reference state is fully characterized by the squared Brunt–Väisälä frequency $N_0^2=N_{T_0}^2+N_{C_0}^2$, where ${N_{T_0}}^2 = - {\varvec{g}} \varvec{\cdot } \nabla T_0$ and ${N_{C_0}}^2 = - {\varvec{g}} \varvec{\cdot } \nabla C_0$ are the thermal and compositional contributions (Monville et al. 2019). A well-mixed (isentropic) fluid is modeled by ${N_0}^2=0$, whereas we must consider ${N_0}^2>0$ for a stably stratified interior. In the rotating reference frame, the dimensionless Boussinesq equations for the velocity ${\varvec{v}}$, the magnetic field ${\varvec{B}}$, the temperature *T* and the mass fraction of light elements *C* are (Jones 2015)

1a $$\begin{aligned} \mathrm {D}_{t} {\varvec{v}} + 2 {\varvec{\varOmega }} \times {\varvec{v}} + {\varvec{f}}_P&= -\nabla P + E \, \nabla ^2 {\varvec{v}} - \chi \, {\varvec{g}} + Le^2 \, (\nabla \times {\varvec{B}} )\times {\varvec{B}} \end{aligned}$$

1b $$\begin{aligned} \partial _t {\varvec{B}}&= \nabla \times ( {\varvec{v}} \times {\varvec{B}}) + E_m \,\nabla ^2 {\varvec{B}}, \end{aligned}$$

1c $$\begin{aligned} \mathrm {D}_t T&= E_T \, \nabla ^2 T - {\varvec{v}} \varvec{\cdot } \nabla T_0, \end{aligned}$$

1d $$\begin{aligned} \mathrm {D}_t C&= E_C \nabla ^2 C - {\varvec{v}} \varvec{\cdot } \nabla C_0, \end{aligned}$$

with the solenoidal conditions $\nabla \varvec{\cdot } {\varvec{v}} = \nabla \varvec{\cdot } {\varvec{B}} = 0$, where ${\varvec{f}}_P = \dot{{\varvec{\varOmega }}} \times {\varvec{r}}$ is the Poincaré force with the position vector ${\varvec{r}} = (x,y,z)^\top$ in Cartesian coordinates, $\mathrm {D}_t = \partial _t + ({\varvec{v}} \varvec{\cdot } \nabla )$ is the material time derivative, $P = p - |{\varvec{\varOmega }} \times {\varvec{r}}|^2/2$ is the reduced pressure (with the dynamical pressure *p*), and $\chi = T + C$ is the density perturbation. We have introduced in Equations (1) several dimensionless numbers, which are defined as specific ratios of the different time scales of the problem.

The Ekman number $E = \nu _f/(\varOmega _0 R_o^2)$ measures the ratio of the rotation time scale $T_\varOmega = \varOmega _0^{-1}$ to the viscous time scale $R_o^2/\nu _f$, the Lehnert number $Le=B_0/(\varOmega _0 R_o \sqrt{\rho _0 \mu })$ the ratio between the Alfvén time scale $T_A = R_o \sqrt{\rho _0 \mu }/B_0$ and $T_\varOmega$, the magnetic Ekman number $E_m = \eta /(\varOmega _0 R_o^2)$ the ratio between $T_\varOmega$ and the time scale of Ohmic diffusion $R_o^2/\eta$, the thermal Ekman number $E_T = \kappa _T/(\varOmega _0 R_o^2)$ the ratio between $T_\varOmega$ and the time scale of thermal diffusion $R_o^2/\kappa _T$, and the compositional Ekman number $E_C = \kappa _C/(\varOmega _0 R_o^2)$ the ratio between $T_\varOmega$ and the time scale of compositional diffusion $R_o^2/\kappa _C$. Typical values of these numbers for the Earth’s core are given in Table 1, together with the ranges numerically accessible in models.

**Table 1:** Typical values of the dimensionless numbers in the Earth’s liquid core (e.g., Jones 2015), and in most numerical models for normal modes (numerical values are much less realistic in direct numerical simulations of the primitive equations). Symbol $^\dagger$: vanishing diffusion only for asymptotic models in full geometries

Dimensionless number	Definition	Earth’s core	Models
Ekman	$E=\nu _f/(\varOmega _0 R_{o}^2)$	$10^{-15}$	$0^\dagger$ or $\ge 10^{-11}$
Lehnert	$Le=B_0/(\varOmega _0 R_o\sqrt{\rho _0 \mu })$	$10^{-4}$	$\ge 10^{-5}$
Magnetic Ekman	$E_m=\eta /(\varOmega _0 R_{o}^2)$	$10^{-9}-10$	$0^\dagger$ or $\ge 10^{-7}$
Thermal Ekman	$E_T=\kappa _T/(\varOmega _0 R_{o}^2)$	$10^{-14}-10^{-13}$	$0^\dagger$ or $\ge 10^{-9}$
Compositional Ekman	$E_C=\kappa _C/(\varOmega _0 R_{o}^2)$	$\le 10^{-17}$	$0^\dagger$ or $\ge 10^{-9}$

Equations (1) are finally supplemented with boundary conditions (BCs) on the CMB and ICB. The velocity field must satisfy the no-penetration BC ${\varvec{v}} \varvec{\cdot } {\varvec{1}}_n = 0$, where ${\varvec{1}}_n$ is the outward unit vector normal to the boundary. For viscous fluids, one must also prescribe additional BCs for the tangential components of the velocity. The two types of BC that are usually considered are the no-slip BC, which requires additionally that ${\varvec{v}} \times {\varvec{1}}_n = {\varvec{0}}$, or the stress-free BC. These two viscous BCs are believed to yield qualitatively similar results in the bulk (Fotheringham and Hollerbach 1998), although the dissipation of the modes could be different (see below). For the temperature and the composition, models usually consider either Dirichlet BCs (e.g., $T =0$) or Neumann BCs (e.g., fixed flux $\nabla T \varvec{\cdot } {\varvec{1}}_n=0$). For the magnetic field, the electrical conductivity of the lowermost mantle (Jault 2015) is usually neglected for normal mode computations. The mantle is thus treated as an electrical insulator, and the magnetic field must satisfy ${\varvec{B}} = \nabla \varPhi _E$ at the CMB, where $\varPhi _E$ is the exterior potential in the mantle. For simplicity, an electrically insulating ICB is also often considered (e.g., Lin and Ogilvie 2018, 2020). However, because such BCs are difficult to enforce in non-spherical geometries, other BCs are sometimes considered (Cébron et al. 2012a).

The above magnetohydrodynamic equations are sufficient to model the liquid core dynamics, as long as the rotational dynamics is known (and imposed) at the CMB and ICB (thus neglecting any feedbacks of the fluid motions on the adjacent layers). However, the outer core is also coupled to the CMB and ICB by various mechanisms (Roberts and Aurnou 2012; Buffett 2015). To account for the interplay with the other layers, one must also consider Equations (5) for the conservation of angular momentum (see Sect. 5 for a coupled model).

### Numerical Methods for the Fluid Modes

Equations (1) admit small-amplitude oscillating solutions, which represent the free modes of the outer core. They are often called Magneto-Archimedean-Coriolis (MAC) modes, due to the combined action of the Coriolis, buoyancy and Lorentz forces.

To compute these modes, it is typically assumed that the outer core dynamics does not modify the (imposed) rotation at the CMB and ICB, and the background convective motions ${\varvec{U}}_0$ of the core are neglected (since they are of smaller amplitude than the solid-body rotation, e.g., Holme 2015). Thus, the fluid core is supposed in co-rotation with the CMB (and the ICB) at the angular velocity ${\varvec{\varOmega }}={\varvec{1}}_z$ in the inertial frame. Then, the MAC modes are formally the small-amplitude perturbations $[{\varvec{u}}, {\varvec{b}}, \varTheta , \xi ]$ that exist upon a quiescent (${\varvec{U}}_0={\varvec{0}}$) and idealized background magnetic field ${\varvec{B}}_0$. The background stratification of the core is left unperturbed. We seek solutions of Equations (1) as

2 $$\begin{aligned}{}[ {\varvec{v}}, {\varvec{B}} ] ({\varvec{r}},t) = [ {\varvec{0}}, {\varvec{B}}_0 ] ({\varvec{r}}) + [ {\varvec{u}}, {\varvec{b}} ] ({\varvec{r}}) \, \mathrm {e}^{\lambda t}, \quad [T, C ] ({\varvec{r}},t) = [ \varTheta , \xi ] ({\varvec{r}}) \, \mathrm {e}^{\lambda t},\end{aligned}$$

with the solenoidal conditions $\nabla \, {\varvec{\cdot }} \, {\varvec{u}} = \nabla \, {\varvec{\cdot }} \, {\varvec{b}} = 0$, and with $\lambda = \sigma + \mathrm {i} \omega$ where $\sigma \in {\mathbb {R}}$ is the damping factor and $\omega \in {\mathbb {R}}$ the angular frequency ($\omega >0$ means that the phase propagation of the normal mode is retrograde). Then, we can linearize Equations (1) to rewrite the problem as a generalized eigenvalue problem (GEP), as we will consider in Sect. 3. Considerable fundamental knowledge about these modes has been recently obtained using numerical computations in Earth-like geometries. To do so, the differential equations are discretized using appropriate numerical techniques, and then the eigenvalue problem is converted into a matrix problem that is solved using available numerical algorithms for dense or sparse matrices.

The majority of numerical studies have considered spherical geometries for simplicity, where the problem can be solved efficiently using a spectral decomposition in latitude and longitude usually in terms of an expansion onto surface spherical harmonics $Y_l^m$ of maximum degree $l\le l_{\max }$ and azimuthal order *m* (with $|m| \le l$). The velocity field (and the magnetic field) is usually sought using the poloidal–toroidal decomposition, though other decompositions can be used (Rieutord 1987, 1991). Due to the orthogonality of the spherical harmonics, the spectral form of the eigenvalue problem can be obtained by projecting the equations onto every spherical harmonic $Y_l^m$, using either symbolic calculus (e.g., Ivers and Phillips 2008) or fast spherical harmonic transformations (as implemented in Schaeffer 2013). This leads to differential equations for the radial scalars that are finally discretized using finite differences (as in Vidal and Schaeffer 2015) or various polynomial expansions that satisfy the appropriate BC, for instance based on Jacobi polynomials (Livermore et al. 2007), Chebyshev polynomials (as in Rieutord and Valdettaro 2018; Lin and Ogilvie 2020), or Gegenbauer polynomials (as in Rekier et al. 2019).

Going beyond the spherical geometry is highly desirable for geophysical applications, since the Earth’s core is not strictly spherical (Koelemeijer 2020). The ellipsoidal geometry has received much attention, since the largest aspherical topographic feature of the CMB is its polar flattening. Unfortunately, the poloidal–toroidal decomposition is not well suited in ellipsoidal geometries, because several important symmetries of the spherical decomposition are not preserved (Ivers 1989). A generalization of the poloidal–toroidal decomposition has been designed in oblate spheroidal coordinates (Schmitt and Jault 2004; Schmitt 2006), but it seems difficult to extend it to triaxial ellipsoids, let alone to shells with arbitrary ellipticities, even if certain families of non-homoeoidal shells (i.e., shells bounded by two similar ellipsoids having a constant ratio of axes) can be tackled with this approach. Hence, considering ellipsoidal geometries is numerically very challenging.

By analogy with the vector spherical harmonics (Rieutord 1987, 1991), one could use the vector ellipsoidal harmonics (which have been recently introduced in Dassios 2012). Yet, a fast numerical algorithm is still lacking to accurately perform the ellipsoidal harmonic transformation, and so this approach has not been considered yet in any numerical models. Alternatively, fully spectral Galerkin descriptions based on global polynomials in the Cartesian coordinates have been developed to solve the diffusionless fluid modes in full ellipsoids (Vantieghem 2014; Vidal et al. 2019, 2020; Gerick et al. 2020; Vidal and Cébron 2020). A wealth of intuition about the fluid modes in non-spherical geometries has built up using the latter approach, but it cannot be used for non-vanishing viscosity (to match the tangential BC of the velocity field), or in ellipsoidal shells. One can overcome this problem in homoeoidal shells by using the Poincaré transformation (i.e., the ellipsoidal volume is remapped onto a computational spherical domain in which distorted equations must be solved, see in (Lorenzani and Tilgner 2001, 2003; Ivers 2017a)). For arbitrary shells, one could use non-orthogonal spherical-like coordinates to solve (Rogister and Rochester 2004; Rochester et al. 2014), or Taylor-expand the non-spherical BC (Rekier et al. 2019; Triana et al. 2019). Non-spectral flexible methods could also be considered (e.g., finite elements (Su et al. 2020)).

Another important issue with non-spherical domains is to enforce the magnetic BC for an electrically conducting mantle, which are global BC (because the magnetic field must match an exterior potential field everywhere at the boundary). Implementing these magnetic BC is very difficult in the ellipsoidal geometry (Ivers 2017b), due to the lack of fast ellipsoidal harmonic transformation for numerical computations. Thus, alternative (local) magnetic BCs are generally considered for the magnetic field in non-spherical geometries (Cébron et al. 2012a).

## Fluid Modes of the Outer Core

We focus here on the properties of the MAC modes. They have been largely investigated in unbounded (or plane-layer) geometries for mathematical simplicity (Finlay 2008), but their properties are strongly modified in the presence of closed boundaries. It is thus important to account for Earth-like geometries for geophysical applications (see §6 ).

**Fig. 2:**
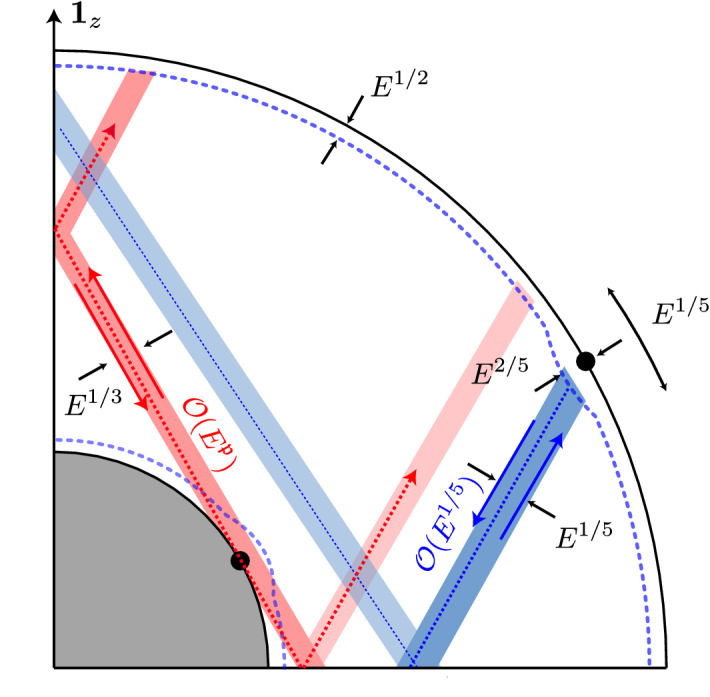
Illustration of viscously driven layers and flows in a spherical shell. Modified from figure 1 in Calkins et al. (2010). The dotted blue lines near the CMB and ICB represent the Ekman layer thickness. The two black dots on the inner and outer boundaries represent the critical colatitudes. Oblique red and blue beams represent oscillatory shear layers resulting from the eruption of the Ekman boundary layer at the critical colatitudes (Kerswell 1995). The scaling laws for the Ekman boundary layer at the ICB are identical to those at the CMB, except for the velocity amplitude in the shear layer $\propto {\mathcal {O}}(E^{\mathfrak {p}})$ where the exponent is still disputed (Kerswell 1995; Le Dizès and Le Bars 2017)

**Fig. 3:**
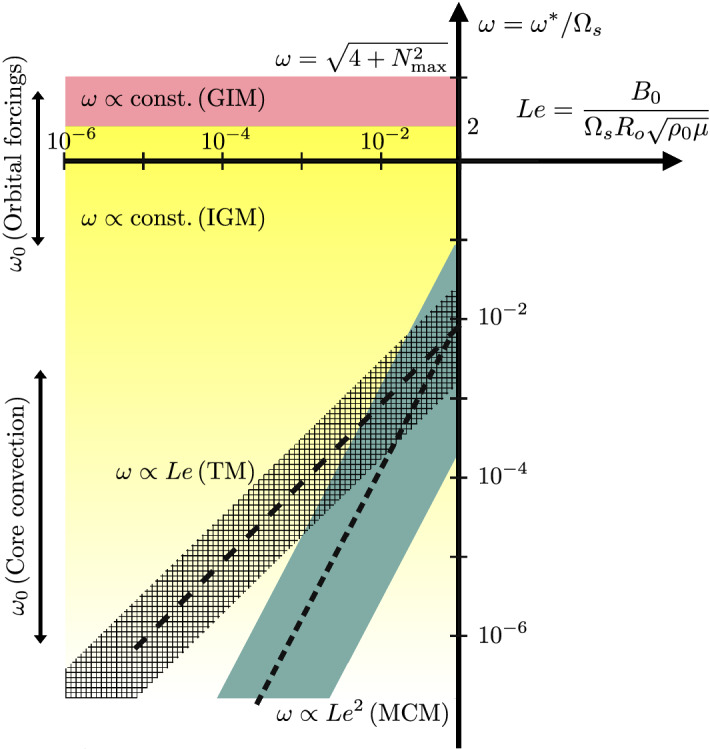
Schematic diagram of the (dimensionless) angular frequency $\omega$ for MAC modes in the outer core, as a function of the Lehnert number *Le*. Adapted from Labbé et al. (2015), Vidal et al. (2019), Gerick et al. (2020), Gerick et al. (2021). GIM: gravito-inertial modes (red area). IGM: inertia-gravity modes (yellow area). Other colored regions illustrate the typical frequency range of the largest-scale magnetic modes, and their scaling law as a function of *Le*. TM: torsional modes (hatched area). MCM: magneto-Coriolis modes (blue area). Typical forcing frequencies $\omega _0$ for orbital forcings and core convection are also indicated (see Sect. 6)

Prior to any computations, it is worth discussing the order of magnitude of the various dimensionless numbers in Table 1, to gain physical insights into the leading order physical effects. The outer core is characterized by very small diffusive effects (compared to rotation), as measured by the very small value of the Ekman number that may suggest that viscosity is not important for the modes at leading order. However, non-vanishing viscosity is responsible for the occurrence of thin viscous layers (see Fig. 2), such as the very thin Ekman boundary layer of typical depth $\propto E^{1/2}$ (Greenspan 1968), which corresponds to approximately 1 m thickness in the Earth’s core with $E=10^{-15}$, or the internal shear layers spawned by the eruption of the Ekman layers at the critical colatitudes (Kerswell 1995). These diffusive layers, although quite thin in the Earth’s core, must be carefully modeled to get realistic predictions for the damping of the modes (Buffett 2010b; Lin and Ogilvie 2020). Moreover, including viscosity and magnetic diffusion is also crucial in shell geometries (as we will discuss below), even if *E* and $E_m$ are very small in the core. Unfortunately, the spatial resolutions needed to resolve these structures require computational resources that are currently well beyond the capabilities of all state-of-the-art codes (see the last column in Table 1). Therefore, one must never forget that some numerical compromises must be taken in numerical models and that our ability to properly extrapolate the results to the core is always somewhat uncertain.

### Diffusionless Modes in Neutrally Buoyant, Full Ellipsoids

We describe in this section the diffusionless modes in a full ellipsoidal geometry (i.e., no inner core), and we also neglect buoyancy effects by setting $\varTheta =\xi =0$ in expansion (2). This canonical setup allows us to discuss the basic properties of the fluid modes (before reintroducing the effects associated with buoyancy and shell geometries in the next subsections). We start our presentation with the non-magnetic modes of a fluid undergoing solid-body rotation, which are sustained by the Coriolis force and are usually referred to as inertial modes (or sometimes Coriolis modes (Ivers 2017a)). These modes have been studied for a long time (Poincaré 1885; Bryan 1889), and they play a fundamental role in rapidly rotating fluids (Greenspan 1968; Zhang and Liao 2017). They are orthogonal solutions of the inertial mode problem (Greenspan 1968)

3 $$\begin{aligned} \lambda \, {\varvec{u}} + 2\, {\varvec{1}}_z \times {\varvec{u}} = - \nabla p, \quad \int _{{\mathcal {V}}} {\varvec{u}}_i^\dagger \varvec{\cdot } {\varvec{u}}_j \, \mathrm {d} {\mathcal {V}} = \Vert {\varvec{u}}_i\Vert \delta _{ij}. \end{aligned}$$

together with purely imaginary eigenvalues $\lambda = \mathrm {i} \omega$ with $|\omega | < 2$. Note that orthogonality (3b) between two inertial modes is valid in any geometry (Greenspan 1968) and also holds for degenerate modes with $\omega _i=\omega _j$ (orthogonal solutions can be constructed using the Gram–Schmidt procedure (Ivers et al. 2015)). The inertial mode problem has also the great feature to admit smooth solutions in Cartesian polynomial vectors that can be obtained analytically in spheroids (Zhang and Liao 2017) or computed numerically in ellipsoids (Vantieghem 2014; Vidal et al. 2020). The inertial mode spectrum admits discrete values, which are dense in the interval $-2< \omega <2$ (Backus and Rieutord 2017; Ivers 2017a). Another very intriguing mathematical property of the inertial modes in ellipsoids is that they form a complete set (like the set of spherical harmonics on spherical surfaces) for any smooth incompressible motion ${\varvec{v}}$. This property is not generic, as it holds only in a few simple geometries (Backus and Rieutord 2017; Ivers 2017a; Zhang and Liao 2017).

The inertial modes of uniform vorticity play a special role. They are sought in the generic form ${\varvec{u}} = {\varvec{w}} \times {\varvec{r}} + \nabla \varPsi$ Noir and Cébron (2013), where $\varPsi$ is a scalar potential introduced to satisfy the no-penetration BC ${\varvec{u}} \varvec{\cdot } {\varvec{1}}_n = 0$ on the CMB. They have a spatially uniform rotation rate $\nabla \times {\varvec{u}} = 2 {\varvec{w}}$ along one of the Cartesian axis. The linear mode with ${\varvec{w}}\propto {\varvec{1}}_z$ is the largest-scale geostrophic mode, whereas the other two, with equatorial rotation rates, are usually called spin-over modes (Vantieghem et al. 2015). These modes are also very important for the rotational dynamics of the Earth. They can be directly excited by several mechanical forcings (e.g., tides, precession or length-of-day variations (Le Bars et al. 2015), involving various forcing frequencies) in the core, and they are the only inertial modes to have nonzero angular momentum in the ellipsoid Ivers (2017a).

While the high-frequency inertial modes are 3-D, two other subsets of modes made of two-dimensional, i.e., nearly or exactly vertically invariant, flows are worth discussing for planetary applications. The subset of degenerate modes with a zero angular frequency ($\omega =0$) is made of geostrophic modes ${\varvec{u}}_{G}$, which individually satisfy the geostrophic balance $2\, {\varvec{1}}_z \times {\varvec{u}}_{G} = - \nabla p_{G}$ such that they are invariant along the rotation axis (i.e., $\partial _z {\varvec{u}}_{G} = {\varvec{0}}$). The other important subset is made of low-frequency inertial modes, which are quasi-geostrophic (i.e., almost invariant along the rotation axis).

We now consider magnetic effects in the $Le \ll 1$ regime, appropriate for Earth. The typical frequency diagram illustrated in Fig. 3, which is based on published numerical computations (Labbé et al. 2015; Vidal et al. 2019; Gerick et al. 2020, 2021), calls for several comments. The high-frequency spectrum is actually barely modified by the Lorentz force in full ellipsoids (because the magnetic field operates on much slower time scales than the rotation period in the core). The corresponding modes are only slightly modified inertial modes. In contrast, the low-frequency spectrum is significantly affected by the Lorentz force. Two families of low-frequency magnetic modes can be identified in Fig. 3, which have very different properties. One can first identify the slow magneto-Coriolis modes (MCM), which are shaped by the combined action of the Lorentz and Coriolis forces (Malkus 1967). These low-frequency modes are characterized by the scaling law $|\omega | \propto Le^2$ (Labbé et al. 2015; Vidal et al. 2019) and have a small kinetic energy compared to the magnetic energy (Acheson and Hide 1973; Gerick et al. 2020). A more quantitative description of the slow magnetic modes is beyond the scope of this review, because they depend on the geometry of ${\varvec{B}}_0$ Schmitt (2010). The other family is the torsional Alfvén modes (TM), displaying the scaling law $|\omega | \propto Le$. They can only exist for background magnetic fields satisfying $\varvec{\varOmega } \times (\nabla \times {\varvec{B}}_0) \ne {\varvec{0}}$ Vidal et al. (2019). Moreover, they have nearly equal kinetic and magnetic energies (Gerick et al. 2020), and their velocity field is mainly geostrophic. A more detailed account of the TM is given below.

### Torsional Alfvén modes

Torsional Alfvén modes (TM) are azimuthal oscillations of rigid cylindrical surfaces (geostrophic cylinders) aligned with the rotation axis. First introduced by Braginsky (1970), they involve the axially symmetric, azimuthal component (or, more simply, the zonal component) of geostrophic flows and result from a balance between their inertial accelerations and a restoring Lorentz force. We present below a short review of TM; a more in-depth treatments of TM can be found in several other articles (e.g., Bloxham 1998; Jault 2003; Dumberry 2009b; Roberts and Aurnou 2012; Jault and Finlay 2015).

In strict terms, the terminology ’geostrophic’ is reserved to describe flows that obey a balance between pressure gradients and the Coriolis force. The accelerating zonal flows involved in TM are then not geostrophic but time-dependent zonal geostrophic flows, or ‘pseudo-geostrophic’ flows Gans (1971).

TM are a subset of the more general family of modes influenced by Lorentz and Coriolis forces (see above). The governing equation for the TM can be constructed by integrating the azimuthal component of the momentum equation on geostrophic cylinders. In non-dimensional form, this gives

4 $$\begin{aligned} \frac{\partial {{\bar{u}}_\phi } }{\partial t} = \frac{Le^2}{4 \pi s H} \int \nabla \cdot (s \mathbf{B}_p b_{\phi }) \, s \mathrm {d}z\mathrm {d}\phi , \end{aligned}$$

where ${\bar{u}}_\phi$ denotes rigid zonal motion of geostrophic cylinders of height *H* at cylindrical radius *s*, and $\mathbf{B}_p$ and $b_{\phi }$ are, respectively, the background quasi-steady poloidal and small azimuthal perturbation of the magnetic field. We have neglected the material acceleration term (assuming small flow amplitude) and viscous forces. The pressure term vanishes identically upon integration and so does the Coriolis term when considering incompressible flows. Assuming that magnetic field perturbations are only induced by ${\bar{u}}_\phi$ and neglecting diffusion, we obtain

5 $$\begin{aligned} \frac{\partial b_{\phi }}{\partial t} = s \mathbf{B}_p \cdot \nabla \xi , \end{aligned}$$

with $\xi (s,t) = {\bar{u}}_\phi /s$. Taking the time-derivative of Equation (2) and substituting Equation (3), we obtain the 1-D torsional mode equation

6 $$\begin{aligned} s^3 H \frac{\partial ^2\xi }{\partial t^2} = \frac{\partial }{\partial s}\left( s^3 H {v_A}^2 \frac{\partial \xi }{\partial s} \right) - \frac{\partial f_\phi }{\partial t}, \quad {v_A}^2(s) = \frac{Le^2}{4\pi s H} \int (B_s)^2 \, s \mathrm {d}z\mathrm {d}\phi ,\end{aligned}$$

where ${v_A}$ is the Alfvén velocity in the *s* direction and $f_\phi$ captures the sum of forces that the top and bottom of the cylinders exert on the solid boundaries of the fluid core. Note that the inertial acceleration is balanced solely by the Lorentz force, a characteristic of Alfvén waves. Note also that it is only the *s*-component of the background magnetic field that is involved in TM; differentially rotating geostrophic cylinders stretch $B_s$ field lines and, as if cylinders were joined by elastic strings, this provides the restoring force for the modes.

The eigenfunctions and frequencies of the TM depend on the spatial variations in ${B_s}$, and also on the strength and nature of the coupling with the mantle and inner core. As such, TM also present an opportunity to illuminate physical quantities inside the core or at the CMB for which we otherwise have little or no information, in particular the strength of the $B_s$-field inside the core (e.g., Zatman and Bloxham 1997; Buffett et al. 2009; Gillet et al. 2010a). The coupling between TM and the mantle and inner core through $f_\phi$ can be from electromagnetic, viscous and topographic forces (although a proper treatment of the latter requires an extension beyond this one-dimensional TM equation (see Gerick et al. 2020)). This coupling allows for an exchange of axial angular momentum between the fluid core (carried by TM), inner core and mantle; the latter two are further coupled by gravitational forces. The changes in the mantle angular velocity can be observed as length-of-day (LOD) changes. Several studies have investigated how TM are affected by different core–mantle coupling scenarios and how they are linked to the observed LOD changes (e.g., Braginsky 1970; Buffett 1998; Jault 2003; Mound and Buffett 2003, 2005, 2007; Dumberry and Mound 2008; Buffett et al. 2009; Dumberry and Mound 2010; Roberts and Aurnou 2012; Gillet et al. 2010a).

It is noteworthy to point out that a paradigm shift occurred in the last decade concerning TM. Whereas the fundamental mode of TM was assumed to have a period of 60-80 yrs in the original study of Braginsky (1970) and in most of the studies mentioned above, it is now generally believed that the period of the fundamental mode is approximately 6 yr Gillet et al. (2010a). To wit, a simple order of magnitude estimate for the period of the fundamental mode is, in dimensional form,

7 $$\begin{aligned} \tau \approx (\varOmega _0 Le)^{-1}\approx R_0 \frac{\sqrt{\rho _0 \mu }}{ |B_s| }. \end{aligned}$$

Using $R_0 = 3.5 \times 10^6$ m, $\rho _0 = 10^4$ kg $\hbox {m}^{-3}$, $\mu = 4 \pi \times 10^{-7}$ N $\hbox {A}^{-2}$, and an estimate of the magnetic field strength within the core of $|B_s| \approx 2$ mT (e.g., Christensen and Aubert 2006), this gives $\tau \approx 6$ years. This simple order-of-magnitude estimate suggests that TM should have periods of a few years. More details on this change in perspective are presented in Sect. 6.3.

A localized perturbation of $\xi$ instigates an Alfvén wave that propagates in the *s*-direction according to Equation (6a). Such waves have been identified in geodynamo simulations (Wicht and Christensen 2010; Teed et al. 2015; Schaeffer et al. 2017; Aubert 2018) and also in simplified models of core dynamics (Gillet et al. 2017; More and Dumberry 2018), further demonstrating that we expect their presence in core flows. A few studies have also investigated the reflection properties of torsional waves at the equator (Schaeffer et al. 2012; Cox et al. 2014; Schaeffer and Jault 2016; Maffei and Jackson 2016; Gillet et al. 2017) and the secular variation they induce (Cox et al. 2016).

### Stratification Effects

**Fig. 4:**
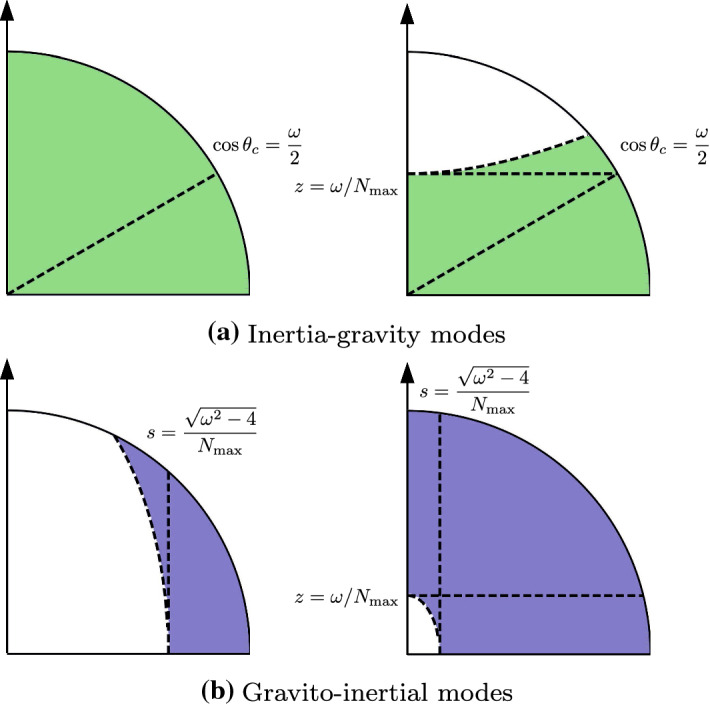
Domains of existence (colored areas) of the inertia-gravity modes in (a) and gravito-inertial modes in (b). Sketch in a meridional plane, where the solid arrow indicates the axis of rotation. Oblique dashed line shows the critical colatitude $\omega = 2 \cos \theta _c$. Horizontal dashed line shows $z=\omega /N_{\max }$. *Top left panel*: Modes ${\mathcal {H}}_1$ with $N_{\max } \le \omega ^2 \le 4$. *Top right panel*: Modes ${\mathcal {H}}_2$ with $0 \le \omega ^2 \le \min (4, N_{\max }^2)$. *Bottom left panel*: Modes ${\mathcal {E}}_1$ with $\max (4, N_{\max }^2) \le \omega ^2 \le 4 + N_{\max }^2$. *Bottom right panel*: Modes ${\mathcal {E}}_2$ with $4 \le \omega ^2 \le N_{\max }^2$

We now reintroduce buoyancy effects to seek the properties of the stratified modes, i.e., modes that occur under the added influence of stable stratification (when $N_0^2 \ge 0$). The outer core may have indeed been stably stratified in density in the past Nimmo (2015), Jacobson et al. (2017), or presently in a thin layer atop the core (see the discussion below). We first set $E_T=E_C=0$ in the temperature and composition equations (1c,1d), because thermal and compositional diffusive effects are expected to be small in the core (see Table 1). Equations 1c and 1d can then be combined into a single equation for the density perturbation $\varTheta + \xi$, which is associated with the background squared Brunt–Väisälä frequency $N_0^2 = N_{T_0}^2 + N_{C_0}^2$ (in dimensionless form). We start with the non-magnetic modes (with $Le=0$), since a general theory has been obtained for arbitrary $N_0^2$ in this case (Friedlander and Siegmann 1982).

We only outline below the most important features of the theory for planetary applications. To do so, we consider that the gravity field varies linearly in radius and assume for simplicity that the Brunt–Väisälä frequency also has a linear variation in radius. We then introduce $N_{\max } = \max (N_0)$, the maximum value of $N_0$ at the CMB, which is the key parameter in the theory. As found for the inertial modes, the non-magnetic stratified modes have purely imaginary eigenvalues $\lambda =\mathrm {i} \omega$ bounded by $\omega ^2 \le N_{\max }^2 + 4$. However, the stratification significantly modifies the spatial structure of the modes as illustrated in Fig. 4. The strength of stratification demarcates regions where the modes can exist (hyperbolic domains) and where the modes are evanescent (elliptic domains).

Different families of modes can be defined according to the shape of the critical surfaces, on which the transition between the two domains occurs. The first family consists of the modes with hyperbolic turning surfaces (see Fig. 4a), which are classified as

8 $$\begin{aligned} {\mathcal {H}}_1 : N_{\max } \le \omega ^2 \le 4, \quad {\mathcal {H}}_2 : 0 \le \omega ^2 \le \min (4, N_{\max }^2),\end{aligned}$$

The family ${\mathcal {H}}_1$ coalesces into almost pure inertial modes when $N_{\max }\ll 1$, so they are often named inertia-gravity modes. The ones belonging to the family ${\mathcal {H}}_1$ can exist within the whole fluid volume (as the pure inertial modes), but for the family ${\mathcal {H}}_2$ the inertia-gravity modes are confined within a smaller hyperboloid volume which is delimited from above and below by the critical colatitudes given by $\cos \theta _c = \omega /2$ (the latter formula is not modified by the stratification). The second family consists of the modes associated with elliptical turning surfaces (see Fig. 4b), which are classified as

9 $$\begin{aligned} {\mathcal {E}}_1 : \max (4, N_{\max }^2) \le \omega ^2 \le 4 + N_{\max }^2, \quad {\mathcal {E}}_2 : 4 \le \omega ^2 \le N_{\max }^2,\end{aligned}$$

In the limit of strong stratification $N_{\max } \gg 2$, the modes of the family ${\mathcal {E}}_2$ approach the pure gravity modes, which exist in the whole stratified volume. Hence, these two families are named gravito-inertial modes. The elliptical turning surfaces can encompass (or not) the rotation axis, depending on the strength of stratification. Finally, note that stratification is expected to have similar effects on the structures of the MAC modes, but only a few magnetic field geometries ${\varvec{B}}_0$ have been investigated (Friedlander 1987, 1989; Buffett and Matsui 2019).

Far-reaching implications can thus be drawn from the aforementioned results. Smooth diffusionless solutions probably exist for the family ${\mathcal {H}}_1$ of inertia-gravity modes, but this is not guaranteed for the other families $[{\mathcal {H}}_2,{\mathcal {E}}_1,{\mathcal {E}}_2]$ in the diffusionless regime due to the presence of the turning surface in the bulk. The mathematical problem for the stratified modes is thus much more difficult than for neutrally buoyant fluids, even without an inner core. Note that the above theory does not strictly assume a full geometry, and is also valid in shells (Dintrans et al. 1999). A strong equatorial trapping of some inertia-gravity modes (see panel right in Fig. 4a) could thus occur in the (possible) outermost stratified layer of the core (see the discussion in Sect. 6).

Finally, one may wonder how the diffusionless modes are modified when double-diffusive effects are retained (i.e., with $E_T \ne E_C$). Inertia-gravity (and gravito-inertial) modes still only exist when $N_0^2 = N_{T_0}^2 + N_{C_0}^2>0$, otherwise the fluid is subject to thermo-compositional convection (Jones 2015), but different situations occur depending on the signs of $N_{T_0}^2$ and $N_{C_0}^2$. If the thermal and compositional background profiles are both stabilizing (i.e., $N_{T_0}^2>0$ and $N_{C_0}^2>0$), then the above theory remains valid in the low-diffusive regime. The situation is more intricate when $N_{T_0}^2$ and $N_{C_0}^2$ have opposite signs. The fluid can undergo double-diffusive convection (DDC), even when $N_0^2>0$ due to double-diffusive effects (Garaud 2018; Monville et al. 2019). DDC is usually referred to as semi-convection when $N_{T_0}^2<0$ and $N_{C_0}^2>0$, whereas it is called finger convection when $N_{T_0}^2>0$ and $N_{C_0}^2<0$. The stratified modes may thus coexist with DDC or be superseded by DDC if the convection forcing is strong enough.

### Introducing an Inner Core and Diffusion

We now discuss the effects of a solid inner core and diffusion on the fluid modes. Contrary to the full ellipsoidal geometry, the mathematical problem is far from being fully understood in shell geometries. This can be evidenced by considering the equation for the pressure perturbation *p*. The inertial mode problem (3) can be recast as the Poincaré equation for the pressure $\lambda ^2 \nabla ^2 p + 4 ({\varvec{1}}_z \varvec{\cdot } \nabla )^2 \, p = 0$ (Zhang and Liao 2017). The Poincaré equation is known to be an ill-posed mathematical problem when associated with appropriate BC (Rieutord et al. 2000), because it is hyperbolic in the whole domain while the pressure must satisfy BC of mixed type (i.e., a relation between *p* and $\nabla p$ on the boundary). Pressure equations of mixed typed can also be obtained for the stratified (Friedlander and Siegmann 1982) and MAC modes (Friedlander 1987, 1989), which are still ill-posed with the supplied BC. Thus, the geometry of the boundaries is crucial to determine the properties of the magnetohydrodynamic modes, and the shell geometry may prevent the existence of smooth diffusionless solutions.

**Fig. 5:**
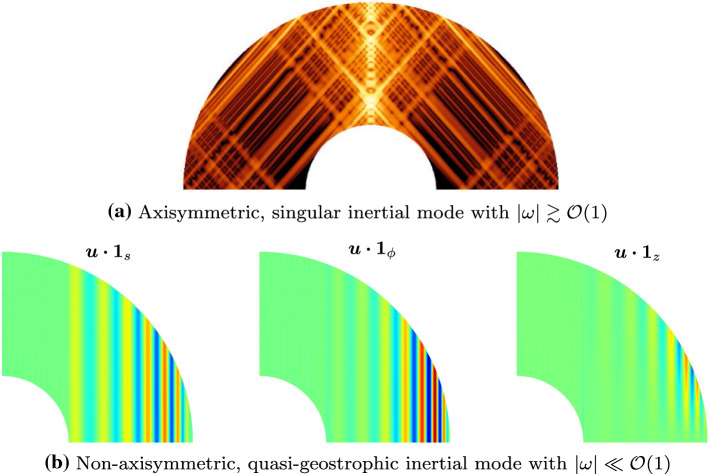
Inertial modes at $E=10^{-8}$ in a spherical shell with ratio $\eta =0.35$, computed with an open-source code (Vidal and Schaeffer 2015). Meridional slices for the local kinetic energy (one-sided, logarithmic scale) in panel (a), and the three cylindrical components of ${\varvec{u}}$ (double-sided, linear scale) in panel (b)

The inertial modes have attracted much attention in shells. The only known analytical solutions in inviscid spherical shells are purely toroidal modes in the form ${\varvec{u}} = \nabla \times ( T_l^m(r) Y_l^m \, {\varvec{r}})$ with $\omega = 2m/(l(l+1))$, and the three uniform-vorticity modes in ellipsoidal shells with identical inner and outer ellipticities. The pathological nature of the non-toroidal solutions of the Poincaré equation in shell geometries was soon conjectured (Stewartson and Rickard 1969) and later confirmed numerically for vanishingly small viscosity in spherical shells (Rieutord 1995; Rieutord and Valdettaro 1997), as illustrated in Fig. 5(a). Various singularities have been identified (see the discussion in Rieutord (2000)), but the most interesting ones are associated with the characteristic curves (or rays) of the Poincaré equation. These curves can converge toward periodic orbits (named attractors) in certain frequency bands or form simple closed trajectories. Viscosity acts to regularize these singularities, which take the form of detached shear layers that closely follow the inviscid attractor patterns (Rieutord and Valdettaro 1997; Rieutord et al. 2001; Rieutord and Valdettaro 2018). These shear layers exist for both the no-slip and stress-free BCs Fotheringham and Hollerbach (1998), showing that they are universal features of the non-toroidal modes in shell geometries. Stress-free BCs suppress for the most part the emergence of viscous boundary layers, thus preventing the internal shear layers associated with the eruptions of the boundary layers at the critical latitudes from appearing. However, even with stress-free BCs, the internal shear layers associated with the characteristic curves (e.g., leading to attractors) or caused by differing ellipticities of the ICB and CMB might still appear (Tilgner 1999). Note that internal shear layers can also be spawned without Ekman layers (when using stress-free boundaries), if the CMB and ICB have different ellipticities (Tilgner 1999).

The presence of internal shear layers is known to affect the damping rate $\sigma \le 0$ of the modes in shell geometries. Taking into account these layers is thus important for planetary applications, since $\sigma$ directly controls the typical lifetime of the modes after their excitation (and so their possible detection). The differences between full and shell geometries can be clearly illustrated by considering the damping rate of the inertial modes. In full ellipsoidal geometries, the damping rate scales as $|\sigma | \propto E^{1/2}$ for no-slip BC (Greenspan (1968), since it is controlled by Ekman boundary layers), or $|\sigma | \propto E$ for stress-free BC Liao et al. (2001). Note that the internal shear layers spawned by the eruption of the Ekman layer at the critical colatitudes (see Fig. 2) have a negligible contribution on the volume-averaged damping rate in full geometries (Hollerbach and Kerswell 1995). However, generic scaling laws are not precisely known in shell geometries when $E\ll 1$. For instance, it has been initially argued (using order-of-magnitude arguments) that the damping rate should scale as $|\sigma | \propto E^{1/2}$ for both no-slip and stress-free BC (Rieutord and Valdettaro 1997) and, for numerical simplicity, only stress-free BC have been considered to numerically explore the regime $E\ge 10^{-10}$ (which is the current limit of state-of-the art models). However, different scaling laws have been numerically reported with stress-free BC at low Ekman numbers (Rieutord and Valdettaro 2018). No-slip BCs have not been considered yet when $E \ll 1$, but no-slip BC could enhance the viscous effects due to the internal shear layers (see (Fotheringham and Hollerbach 1998) for $E\ge 10^{-6.5}$). Thus, the asymptotic behavior of $\sigma$ in shell geometries remains an open question when $E\ll 1$. The scaling laws for the damping rates of the MAC modes are even more speculative, because thermal and Ohmic effects have also to be accounted for. For instance, fast inertial modes displaying attractor patterns could be strongly modified by Ohmic diffusion when $Le > {\mathcal {O}}(E_m^{2/3})$ (Lin and Ogilvie 2018), which may apply to the Earth’s core.

Note that singular modes naturally exist in stably stratified fluids (Rieutord and Noui 1999; Dintrans et al. 1999; Mirouh et al. 2016), but the 3-D magnetic modes have received scant attention so far in shell geometries. One may speculate on the existence in planetary conditions of some singular MC modes, especially those possibly exhibiting equatorially antisymmetric velocity or magnetic fields (Schmitt 2010), but such modes remain to be investigated numerically. Diffusive magnetic modes dominated by the diffusion term in Equation (1b), which are reminiscent of the free-decay magnetic modes (Moffatt and Dormy 2019) and disappear when $E_m=0$, have also been examined (Schmitt 2012), but their relevance to planets is unclear.

A few points are now worth summarizing in the context of planetary applications. The presence of singularities in the mathematical problem when $E=0$ means that the limit $E\rightarrow 0$ is singular. Inviscid models cannot rigorously represent the Earth’s core, which is characterized by the very small but nonzero value $E=10^{-15}$. Considering inviscid models in shells also raises concerns about numerical convergence of the solutions, because the mathematical singularities can only be regularized by the numerical resolution acting as a strong (inconsistent) numerical diffusion in the bulk (which varies with the considered resolution). Diffusionless numerical results cannot be numerically converged and are expected to drastically change if the resolution were further increased. The situation is even worse for inviscid models in non-homoeoidal geometries, because additional shear layers are triggered due to ellipticity effects (even without Ekman layers, as explained in (Tilgner 1999)). Discarding viscosity is also a poor approach to get realistic predictions for the toroidal modes, because the internal shear layers are believed to play an important role in the dissipation in shells (e.g., Buffett (2010b), Lin and Ogilvie (2020), contrary to full geometries). For the aforementioned reasons, one should strive to include physical diffusion in shell geometries, and a particular attention must then be paid to numerical convergence (which is difficult to obtain when $E \ll 1$ (Valdettaro et al. 2007; Rieutord and Valdettaro 2018)).

Finally, the existence of singular modes suggests that the spectrum of MAC modes in shell geometries could be almost empty in the asymptotic regime of vanishing diffusion. However, it does not completely preclude the existence of some smooth modes in the shell (but only if diffusion is included). For instance, it has been shown that some inertial modes with simple structure in the direction of the axis of rotation and sufficiently small azimuthal wave number *m*, which are trapped in the equatorial region, are only weakly modified by the presence of an inner core (Zhang 1992, 1993). Large-scale almost regular modes have also been reported in experimental configurations at moderate values of *E* (Barik et al. 2018). Finally, some low-frequency QG inertial modes, even with possibly small wavelength along the cylindrical radius, can also be weakly sensitive to an inner core as illustrated in Fig. 5(b). The latter observation may suggest that asymptotic models of QG modes could reasonably be used in shells.

## Rotational Modes of the Global Earth

We describe in this section an additional set of free modes specific to oblate planetary bodies comprised of a mantle, fluid core and inner core. These modes, associated with independent precessions of the three constituting layers, are especially interesting because their frequencies are embedded within the spectrum of core-specific modes discussed so far. In a simplified picture, these are captured by a set of three equations (often referred to as the Liouville equations) describing the evolution of the angular momentum of the whole body (${\varvec{H}}$), the fluid core (${\varvec{H}}_f$) and the inner core (${\varvec{H}}_s$). A fourth equation is required to specify the orientation (or tilt, ${\varvec{n}}_s$) of the inner core relative to the mantle. We define a reference frame attached to the mantle, with unit vector ${\varvec{1}}_z$ pointing in the direction of its (undeformed) polar moment of inertia. The instantaneous angular velocity vector of the mantle is ${\varvec{\varOmega }}_m =\varOmega _o {\varvec{1}}_z + {\varvec{\omega }}_m$, where ${\varvec{\omega }}_m$ is a perturbation with respect to the time-averaged rotation at frequency $\varOmega _o$ about ${\varvec{1}}_z$. In the rotating mantle frame, the four equations are Mathews et al. (2002), Dehant and Mathews (2015)

10a $$\begin{aligned}&\mathrm {d}_t {\varvec{H}} + {\varvec{\varOmega }}_m \times {\varvec{H}} = {\varvec{0}}, \end{aligned}$$

10b $$\begin{aligned}&\mathrm {d}_t {\varvec{H}}_f - {\varvec{\omega }}_f \times {\varvec{H}}_f = - {\varvec{\varGamma }}_\text {CMB} - {\varvec{\varGamma }}_\text {ICB}, \end{aligned}$$

10c $$\begin{aligned}&\mathrm {d}_t {\varvec{H}}_s + {\varvec{\varOmega }}_m \times {\varvec{H}}_s = {\varvec{\varGamma }}_s + {\varvec{\varGamma }}_\text {ICB}, \end{aligned}$$

10d $$\begin{aligned}&\mathrm {d}_t {\varvec{n}}_s = {\varvec{\omega }}_s \times {\varvec{1}}_z. \end{aligned}$$

In these equations, $\mathrm {d}_t=\mathrm {d}/\mathrm {d}t$ is the time derivative in the mantle frame, ${\varvec{\omega }}_f$ and ${\varvec{\omega }}_s$ are the differential angular velocities of the fluid core and inner core, both defined as perturbations with respect to ${\varvec{\varOmega }}_m$, ${\varvec{ \varGamma }}_{s}$ is the torque from pressure and self-gravitation exerted on the inner core, ${\varvec{ \varGamma }}_\text {CMB}$ and ${\varvec{\varGamma }}_\text {ICB}$ are the additional torques on the fluid core (at the CMB) and on the inner core (at the ICB), arising from topographic, electromagnetic, and viscous coupling.

In Equations (5), only the rigidly rotating parts of ${\varvec{\omega }}_m$, ${\varvec{\omega }}_f$ and ${\varvec{\omega }}_s$ are tracked. The total flow in the fluid core includes the deviation from rigid rotation that is necessary to satisfy the boundary conditions at the CMB and ICB, but the decomposition is specified such that the total angular momentum of the fluid core at every instant resides in the differential rotation ${\varvec{\omega }}_f$. For this reason, computing the free modes of precession based on Equations (5) is referred to as the angular momentum approach. Not explicitly tracking the non-rigid motion in the fluid core is an approximation, as previous sections of this review clearly indicate that it can host a rich spectrum of free modes. Non-rigid motion likewise occurs in the mantle and inner core, notably from viscoelastic deformation. Although these can be incorporated as corrections to the moments of inertia of each region (e.g., Sasao et al. 1980; Mathews et al. 1991a, b; Buffett et al. 1993), to better take into account non-rigid motion, several studies have sought to improve upon the above angular momentum approach by explicitly solving the set of elastic-gravitational equations within each region (e.g., Smith 1977; Wahr 1981; de Vries and Wahr 1991; Schastok 1997; Rogister 2001; Rochester and Crossley 2009; Rochester et al. 2014). However, the advantage of the system of Equations (5) resides in its simplicity. Furthermore, the motion within the mantle and inner core associated with free precession modes is well approximated by a rigid rotation. Hence, for the purpose of this review, focused on the fluid core, this simplified system provides an adequate starting point.

Four internal modes of precession emerge from Equations (5). These are the Chandler wobble (CW), free core nutation (FCN), free inner core nutation (FICN) and inner core wobble (ICW), e.g., Mathews et al. (1991a, 1991b). The CW, also known as the Eulerian wobble, consists in a prograde motion of the rotation vector ${\varvec{\varOmega }}_m$ around ${\varvec{1}}_z$. The precession motion is sustained by the gyroscopic torque induced by the misalignment of ${\varvec{\varOmega }}_m$ with the oblate geometric figure of the planetary body. The FCN is characterized by a misalignment of the rotation vector of the fluid core with respect to the oblate elliptical shape of the CMB, resulting in a latitudinal pressure gradient on the CMB. The torque from this pressure gradient is referred to alternately as the pressure torque, inertial torque or centrifugal torque. The gyroscopic effect from this torque leads to a retrograde precession of the rotation vector of the core with respect to the mantle frame. The FICN describes the free precession motion of the combined tilted rotation and figure axes of the inner core. As is the case for the FCN, the precession motion is maintained by the pressure torque, here acting on the oblate elliptical figure of the ICB, but the gravitational torque acting to realign the inner core with the mantle also contributes. Finally, the ICW consists in a prograde precession of the tilted oblate figure of the inner core around ${\varvec{1}}_z$. In contrast to the FICN, it is only the figure of the inner core which is tilted, its rotation vector remaining in alignment with ${\varvec{\varOmega }}_m$. As for the CW, the ICW is sustained by the gyroscopic torque induced by the misalignment between its rotation axis and oblate figure, and for this reason it is sometimes referred to as the Chandler wobble of the inner core (Smith 1977). However, the precession rate of the ICW is also influenced by the gravitational torque from the rest of the planet and the pressure torque at the ICB acting on its tilted oblate figure.

All four rotational modes owe their existence to the oblate ellipsoid figures of the mantle, fluid core and inner core. This is in contrast to the MAC modes in the core introduced in Sects. 2 and 3, which exist in spherically shaped fluid core, though they may be modified by ellipsoidal boundaries.

An additional rotational mode is introduced when the angular momentum dynamics is described with respect to a frame rotating about a fixed axis in inertial space as opposed to a mantle-fixed frame that evolves with time as we have used in Equations (5). This is the tilt-over mode (TOM). It consists in a rigid rotation of the whole planet with a period of exactly one sidereal day about an axis that is inclined with respect to that chosen as the reference frame. In this sense, the TOM arises as an artifact of the chosen reference frame. In contrast to the other four modes identified above, the TOM is independent of the interior structure.

Also note that we have neglected in Equations (5) the external gravitational torque such as that from a star (planet) around which a planet (moon) orbits. While the influence of this torque on the internal modes for Earth is negligible, for bodies in synchronous rotation or with a rotation period approaching the orbital period, the free precession modes are altered by the external torque, e.g., Varadi et al. (2005), Baland et al. (2019), Dumberry (2021). Lastly, we have also neglected the influence of surface fluid layers such as oceans and the atmosphere. However, for moons with a global subsurface ocean covered by an icy shell, the above system can be used with the ocean layer taking the role of the fluid core, e.g., Baland et al. (2019).

The interest in these free modes of rotation resides in the fact that their periods depend, primarily, on the internal density structure and, secondarily, on other internal parameters for which observations are scant. Hence, if detected, their periods can be used to constrain the internal structure and dynamics of a planetary body. For the focus of this review article, two modes are of particular interest, the FCN and FICN. For Earth, which is nearly an axisymmetric body, we can specify the polar and mean equatorial moments of inertia of the mantle (subscript *m*), fluid core (subscript *f*) and inner core (subscript *s*) as $(C_m, A_m)$, $(C_f, A_f)$ and $(C_s, A_s)$. The (dimensional) FCN and FICN frequencies as seen in the rotating mantle frame, $\omega _\text {FCN}$ and $\omega _\text {FICN}$, can be written as

11 $$\begin{aligned} \omega _\text {FCN} = -\varOmega _o + \nu _\text {FCN}, \quad \omega _\text {FICN} = -\varOmega _o + \nu _\text {FICN}, \end{aligned}$$

where $\nu _\text {FCN}$ and $\nu _\text {FICN}$ express the same frequencies with respect to a space-fixed frame. Note that in contrast to the convention used for MAC modes (see section 2.2), the negative sign indicates a retrograde precession motion, the usual convention in the literature on this topic. $\nu _\text {FCN}$ and $\nu _\text {FICN}$ are closely approximated by

12a $$\begin{aligned} \nu _\text {FCN}&= - \varOmega _o \left( 1 + \frac{A_f}{A_m} \right) \left( e_f - S_f + K_\text {CMB} + K_\text {ICB}\frac{A_s}{A_f} + {{{\mathcal {D}}}}_\text {FCN} \right) , \end{aligned}$$

12b $$\begin{aligned} \nu _\text {FICN}&= \varOmega _o \left( 1 + \frac{A_s}{A_m} \right) \left( \alpha _2 ( e_s + S_s ) - K_\text {ICB} + {{{\mathcal {D}}}}_\text {FICN} \right) , \end{aligned}$$

where $e_f=(C_f-A_f)/A_f$ and $e_s = (C_s-A_s)/A_s$ are the dynamical ellipticities of the outer and inner cores and $\alpha _2$ captures the combined pressure and gravitational torques acting on a tilted inner core. The additional parameters that enter Equations (12a,b) are complex-valued, namely $[S_f,S_s]$, which are compliances related to global viscoelastic deformation, $[K_\text {CMB},K_\text {ICB}]$, which capture the coupling due to tractions at the CMB and ICB, and $[{{{\mathcal {D}}}}_\text {CMB},\mathcal{D}_\text {ICB}]$, which account for dissipation in the volume of the fluid core. The imaginary part of these parameters maps into the damping rate of the FCN and FICN modes.

Since $e_f$ and $e_s$ are of the order of $\sim 1/400,$ e.g., Mathews et al. (1991b), and other parameters are of similar magnitude or smaller, $|\nu _\text {FCN}| \ll |\omega _\text {FCN}|$ and $|\nu _\text {FICN}| \ll |\omega _\text {FICN}|$. When viewed in the rotating mantle frame, the FCN, FICN, are retrograde precessions with periods close to one day. The amplitude of the daily forced nutations driven by external torques from the Moon, the Sun and other planets is resonantly amplified by these modes, e.g., Dehant et al. (2017). The level of amplification depends on their periods, and hence, this gives constraints on the set of parameters that enter Equations (12), e.g., Koot et al. (2010), Zhu et al. (2017). The response of the Earth to these diurnal torques includes small but observable out-of-phase components, and these reflect dissipation mechanisms connected to the imaginary parts of $\nu _\text {FCN}$ and $\nu _\text {FICN}$.

Because their periods fall within the diurnal band of inertial modes, the latter can influence the morphology, period and attenuation of the FCN and FICN. To properly describe these modes, a more general description of fluid motions than a simple tilted uniform rotation must be adopted and progress in this direction is reviewed in subsequent sections. Moreover, by only tracking the rigid rotation part of the flow, the dissipation within the volume cannot be computed, and ${{{\mathcal {D}}}}_\text {CMB}$ and $\mathcal{D}_\text {ICB}$ are identically zero. Dissipation must then be assigned by the remaining parameters, either in $S_f$ and $S_s$ from viscous deformation of the solid Earth, e.g., Greff-Lefftz et al. (2000), Koot and Dumberry (2011) or into $K_\text {CMB}$ and $K_\text {ICB}$ from viscous and electromagnetic coupling at the fluid core boundaries, e.g., Buffett (1992), Buffett et al. (2002), Mathews and Guo (2005), Deleplace and Cardin (2006), Koot et al. (2010), Koot and Dumberry (2013). While the latter provides constraints for instance on the strength of the radial magnetic field threading the boundaries, the parameters extracted from such an exercise may be incorrect if a significant amount of viscous and/or Ohmic dissipation takes place instead within the volume of the fluid core (through $\mathcal{D}_\text {CMB}$ and ${{{\mathcal {D}}}}_\text {ICB}$) via the excitation of inertial waves.

The ICW is also connected to core flows. The period of the ICW, as seen in the rotating frame, is expected to be in the range of 6-9 yr, e.g., Mathews et al. (1991b), Rochester and Crossley (2009), Dumberry (2009a), Ding et al. (2019), depending on the specific Earth model. This mode has not received the same level of attention as the FCN and the FICN, in large part because its period falls outside the resonance band of forced nutations. However, the wobbling motion of the ICB must necessarily be accompanied by fluid motion other than a simple rigid rotation, e.g., Rogister (2010), Busse (1970). Given the longer period of the ICW, quasi-geostrophic motions are expected, e.g., Seyed-Mahmoud and Rogister (2021). The reported detection of the ICW by Ding et al. (2019) should spur a renewed interest in properly accounting for the fluid motion associated with this mode.

Likewise, at the 14-month period of the CW, core flows involved in this rotational mode are expected to be quasi-geostrophic, e.g., Seyed-Mahmoud and Rogister (2021). However, the excitation, period and damping of the CW are predominantly determined by attributes of the mantle and surface fluid layers. The very weak sensitivity of the CW to the specific geometry of core flows implies that, in contrast to the ICW, the observed CW is less useful to extract information about core processes.

We close this section with a few words on one additional global mode of oscillation between the different layers of a planet, the mantle-inner core gravitational (MICG) mode, which consists in a longitudinal oscillation between the inner core and mantle sustained by the gravitational torque on their misaligned equatorial elliptical figures, e.g., Buffett (1996), Mound and Buffett (2003), Dumberry and Mound (2010). For Earth, the period of the MICG mode is expected to be in the range of 6 to 20 yr, e.g., Mound and Buffett (2006), Davies et al. (2014). At such a period, electromagnetic coupling should prevent any large differential motion at the ICB, e.g., Gubbins (1981). Hence, the MICG mode is necessarily coupled to axisymmetric longitudinal core flows—or more simply, zonal flows—with interannual to decadal timescales that carry axial angular momentum changes and induce length-of-day changes, e.g., Jault et al. (1988), Gillet et al. (2010b). Torsional Alfvén modes are an example of such flows (see Sect. 3.2).

## Coupling Between Inertial and Rotational Modes

The frequencies of the four rotational eigenmodes discussed in the preceding section are within the inertial mode frequency band (i.e., from zero to two times the Earth’s rotation frequency). Thus, the composite mode spectrum comprising the set of rotational modes *and* the set of inertial modes needs to be taken into account when considering the Earth’s response to the various forcings acting on it Smith (1977), Wahr (1981). The composite spectrum is, however, not a simple superposition of the two sets. For instance, one important inertial eigenmode, the so-called *spin-over* mode (not to be confused with the *tilt*-over mode), transforms into the FCN mode if the mantle’s motion is allowed to react to torques exerted by the fluid. In the angular momentum approach, to solve for the rotational modes presented in Sect. 4, the core flow is limited to a tilted uniform vorticity vector and thus unable to represent properly inertial eigenmodes with similar frequencies as the rotational eigenmodes. A strict adoption of this approach is then a poor approximation if mode–mode interactions take place or if additional flow components intervene in the damping of the modes. Without these crucial ingredients, solving the corresponding equation of motion (i.e., the linear momentum approach) does not recover the spectrum of inertial modes (Smith 1977), nor gives satisfactory numerical solutions of the core flow associated with the rotational modes (Seyed-Mahmoud et al. 2017; Rogister and Valette 2009) even when increasing the numerical resolution and allowing for smaller-scale flows.

Considering a diffusionless fluid in a spherical or spheroidal shell is indeed conceptually unsatisfactory due to the general ill-posedness of the wave equation in shell geometries (as discussed in Sect. 3). The ill-posed character of the mathematical problem remains unchanged when the Poincaré force (modeling the unsteady mantle) is included in the momentum equation Rekier et al. (2020). Thus, unless the core flow associated with a given rotational eigenmode happens to be toroidal in a spherical shell (e.g., the FCN, which is regular in incompressible fluids), numerical convergence is not ensured. We note that a solution of an eigenvalue problem is not necessarily converged when the eigenvalue merely appears to reach a stable value as the truncation level is increased. To ensure proper numerical convergence of a solution, the amplitude of each term in the truncated series expansion representing the eigenvector should decrease as higher orders in the expansion are considered. The reported lack of convergence for the FICN Seyed-Mahmoud et al. (2017), Seyed-Mahmoud and Rogister (2021), and the rather puzzling solutions of singular inertial modes presented in Kamruzzaman and Seyed-Mahmoud (2020) (see their figure 5) are likely a manifestation of this problem (see also in subSect. 3.4).

In the following, we discuss the salient features of a 2-layer planet model with a fully fluid core (to avoid the singularities caused by the introduction of a solid inner core in the inviscid case). Since there is no inner core, only two rotational eigenmodes are present, the FCN and the CW, in addition to the inertial eigenmodes of the core. We contrast the viscous case against the inviscid one. The eigenvalues are complex in the former and purely real in the latter, which leads to fundamentally different behavior when two eigenmodes have very similar frequencies.

### Inviscid Core Case

**Fig. 6:**
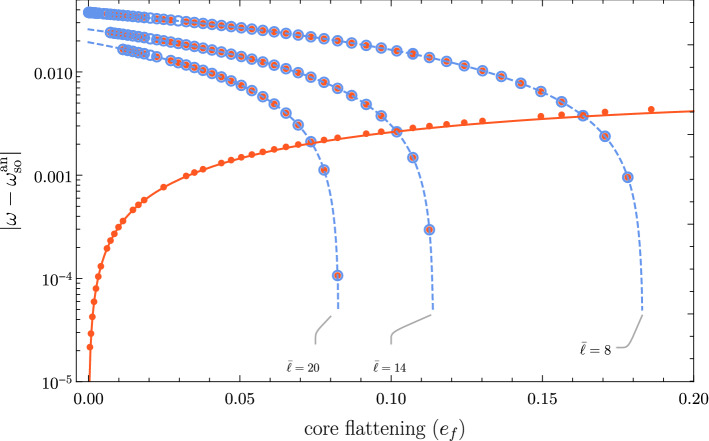
Here the vertical axis represents the difference between the theoretical spin-over frequency $\omega _\mathrm {so}^\mathrm {an}$ and the frequencies $\omega$ of nearby eigenmodes (characterized by their angular wavenumber ${{\bar{\ell }}}$). Red dots correspond to numerical eigenfrequencies computed when the mantle is free to wobble. Open blue circles represent numerical eigenfrequencies when the mantle rotates *uniformly*. The FCN frequency (continuous red line) converges to the spin-over frequency in a planet with a spherical CMB. Other inertial mode frequencies remain unaltered when the mantle is free to wobble (i.e., open blue circles have a matching red dot)

An inviscid fully fluid core can couple to the mantle via pressure torques if the CMB is not spherical. The pioneering work of Hough (Hough 1895) revealed that only uniform vorticity flows are able to exert these torques in a triaxial ellipsoid. This means that the spin-over mode is the only inertial mode that suffers a modification if the mantle is free to wobble. The remaining inertial mode spectrum remains unaltered. Hough’s analysis is valid only up to first-order deviations from a spherical CMB, however. Analytical solutions for the FCN valid to all orders in the flattening of the CMB were obtained only very recently Rekier et al. (2020). In this model, the FCN frequency is computed from Eqs. (5) together with the vorticity equation for the flow. Figure 6 exhibits a couple of important features of these solutions as the flattening of the CMB is varied. The vertical axis in the figure represents the difference between the theoretical spin-over frequency and various other eigenmodes. Red dots correspond to numerical eigenfrequencies computed when the mantle is free to wobble. Open blue circles represent numerical eigenfrequencies when the mantle rotates *uniformly*. They are in excellent agreement with the analytical solutions of Rekier et. al Rekier et al. (2020). The red continuous line and blue dashed line correspond to the analytical FCN and the analytical uniform-rotation inertial modes, respectively.

The FCN frequency depends strongly on the ratio of the Earth’s moments of inertia, whereas the frequencies of the other inertial modes are independent of it. In consequence, for some values of the moments of inertia (controlled by the dynamical flattening $e_f$), the FCN frequency may accidentally coincide with that of another mode. Note that the eigenfrequencies in this model are real; no damping of any sort is considered.

### Viscous Core Case

Viscous effects have a profound impact on the dynamics. The mantle is then subject to viscous torques in addition to pressure torques. The presence of viscosity also leads to the appearance of Ekman boundary layers, which present a limitation for the Taylor expansion of the CMB shape technique (Triana et al. 2019). Such technique relies on a spherical harmonic expansion of the shape of the CMB, and it is valid as long as the (dimensionless) thickness of the boundary layer is not smaller than the CMB’s polar flattening. This clearly precludes the Earth’s case, unfortunately, since the thickness of the boundary layer is determined by the Ekman number *E*. However, this model still provides valuable insight to understand at least qualitatively the interplay between inertial and rotational modes.

There are no known analytical solutions when viscosity is included, so we rely on numerical solutions. To solve simultaneously the fluid dynamical equations for the core and the angular momentum equations for the mantle (i.e., the Euler–Liouville equations), we need to compute the torques as functions of the fluid core velocity. If we choose the mantle as the reference frame for the Navier–Stokes equations, the non-steady motion of the mantle as seen from an inertial frame can be accounted for by introducing the Poincaré force. The eigenmodes are damped in general (i.e., the real part of the eigenvalue is negative) due to the viscous dissipation taking place mostly at the boundaries, although some viscous dissipation also takes place in the bulk of the fluid. However, an important contribution to the damping also comes from the work of the Poincaré force. It can be shown that the damping factor $\sigma$ satisfies a balance of power (Triana et al. 2019), which reads in dimensionless units

13 $$\begin{aligned} 2\,\sigma \,K_f = {\mathcal {D}} - \left( \partial _t {\varvec{M}} \right) \varvec{\cdot } {\varvec{L}}, \end{aligned}$$

where $K_f$ is the total kinetic energy of the fluid, ${\mathcal {D}}$ is the total viscous dissipation, ${\varvec{L}}$ is the total angular momentum of the fluid, and ${\varvec{M}} = {\varvec{\varOmega }} - {{\varvec{1}}_z}$. The quantity $\left( \partial _t {\varvec{M}}\right) \varvec{\cdot } {\varvec{L}}$ is the work associated with the Poincaré force. Note that in a different reference frame than the one attached to the mantle the Poincaré force might be different or nonexistent. In an inertial frame for instance, the Poincaré force vanishes; the equivalent work is then done by stresses originating at the moving boundaries of the fluid.

**Fig. 7:**
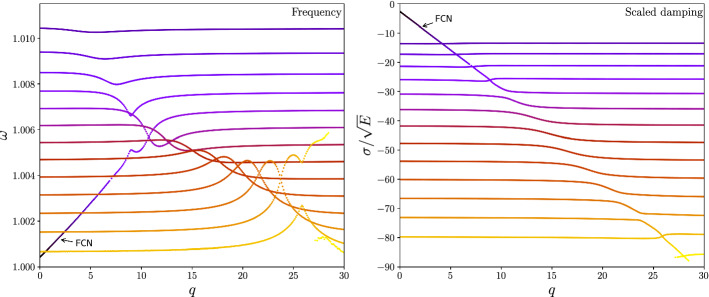
Frequency and damping of nearly diurnal eigenmodes as the parameter *q* is varied. The FCN’s frequency crosses over nearby inertial eigenmodes when $q\ll 1$ until its damping becomes too close to the damping of the modes it is crossing (around $q\sim 9$) and an avoided crossing takes place. The $q=0$ case reduces to the spectrum of inertial modes in *uniformly* rotating planets

Another rather peculiar aspect in the viscous case is that as the mantle’s moment of inertia is varied, when two given eigenvalues get closer to each other in the complex plane the modes seem to avoid crossing each other, either in the real or the imaginary axis. This is illustrated in Fig. 7 where the damping and frequency (i.e., real and imaginary parts of the eigenvalues, respectively) of some eigenmodes with nearly diurnal frequency are plotted as a function of the parameter $q\equiv (\rho _f/\rho _m)/\left( r_0^5-1\right)$, where $\rho _f/\rho _m$ is the core/mantle density ratio and $r_0=R_\mathrm {mantle}/R_\mathrm {cmb}$ is the ratio between the mean outer radius of the mantle and the mean radius of the CMB. The parameter *q* represents essentially the inverse of the mantle’s mean moment of inertia. The CW mode (not shown) does not exhibit any avoided crossings mainly because, in this idealized 2-layer model, its damping is very weak compared to that of the inertial eigenmodes with similar frequencies. Although the model we have just described involves pressure torques, they do not play an essential role in avoided crossings; a spherical model with only viscous torques acting reveals very similar behavior. Note that the FCN in the inviscid case described earlier can have accidental degeneracies with other modes without further consequences, i.e., no avoided crossing takes place. However, inviscid models can exhibit avoided crossings, e.g., when density stratification (Rogister and Valette 2009) or magnetic fields (Gerick et al. (2020) are included.

It is important to note that the frequency (i.e., the imaginary part of the eigenvalue) of the FCN is not altered much by the presence of viscosity. The flattening of the CMB is the most important factor determining it. In this respect, the assumption of a uniform vorticity core flow is adequate.

There is a striking similarity between the FCN’s dynamics we have just described, and the behavior of the viscous spin-over mode in a uniformly rotating spheroid (as in Schmitt 2006). The spin-over mode frequency in that study changes as the flattening of the rigid boundary is increased, becoming closer to the frequencies of the neighboring eigenmodes. Eventually an avoided crossing develops and the modes involved happen to be the *exact* counterpart of the modes involved in the coupled model described above. Thus, in a loose sense, increasing the flattening of a uniformly rotating spheroid is analogous to reducing the mantle’s moment of inertia in a fully coupled inertial-rotational model. We discuss the potential implications for the Earth further below.

## Geophysical Discussion

### Detection of Inertial Modes

The excitation of inertial modes in the outer core (different from the FCN) is conceivable, because they could be excited by several mechanisms in planetary cores (e.g., by orbital forcings when the CMB and ICB have different ellipticities (Tilgner 1999; Ogilvie 2013; Lin and Ogilvie 2017; Lin and Noir 2020), by convection, or earthquakes). There was in fact a claim of detection of inertial modes after analysis of superconducting gravimetry data recorded in Brussels (Belgium) during the strong 1983 Hindukush earthquake Aldridge and Lumb (1987), Melchior and Ducarme (1986). However, data from other recording stations (Zürn et al. (1987) or other earthquakes (Melchior et al. (1988) failed to recover the same resonant peaks. To date, no unequivocal observations of inertial modes have been reported. Low-frequency inertial modes (i.e., interannual and longer) are also unlikely to be revealed by gravimetric data (Gillet et al. 2021). There is a chance, however, that equatorially trapped MAC modes, if their amplitude is large enough, might be observable by space borne magnetic measurements ((Vidal and Schaeffer 2015), e.g., with ESA’s SWARM mission). Also speculatively, there are still stubborn residuals in the observed nutation series from VLBI data that might be caused by the FCN mode interacting in some way with nearby inertial modes. Whether this is actually the case or not cannot be answered yet with our current models. The eventual observation of inertial modes, although unlikely, would be welcomed because it could bring complementary information about the properties (or dynamics) of the core. For instance, the fluid modes could be used to further constrain the stratification or viscosity of the outer core, which are difficult to estimate with seismology (Souriau and Calvet 2015).

For a more in-depth discussion on the measurement of the Earth’s orientation and associated techniques, encompassing nutations and length-of-day variations, we refer the reader to the paper by Rekier et al. in this Special Issue of the journal.

### Effects of a Stable Stratification Atop the Core

The direct observation of bulk inertial modes could be hampered by a possible outermost stably stratified layer, whose existence has been postulated for a long time (Braginsky 1993). We have indeed seen in §3.1 that a stable stratification acts in damping the radial motions (and also can strongly impact the spatial pattern of some inertial modes). Such a stable stratification below the CMB may thus strongly reduce our capability to detect bulk inertial modes in geophysical data, in weakening the associated core flow signature at the CMB (which are used to interpret geomagnetic or geodetic signals, e.g., Holme 2015; Gillet et al. 2021). Much effort has been dedicated to assess the existence of this layer atop the outer core in seismology (e.g., Souriau and Calvet 2015; Tanaka 2007; Helffrich and Kaneshima 2010), geomagnetism (Gubbins 2007; Buffett 2014; Buffett et al. 2016), using nutations (Buffett 2010a), or even with geodynamo models (e.g., Olson et al. 2017; Yan and Stanley 2018; Christensen 2018; Gastine et al. 2020). A strong consensus has not yet been reached on the specific attributes of this stratified layer (thickness, buoyancy frequency), and different plausible scenarios have been proposed to explain its origin.

The interplay between an outer thermal stratification and inertial modes has been explored numerically in spherical shells, with a neutrally buoyant bulk and reasonable values for the amplitude of the stratification in the outer stratified layer. It has been shown that stratification has very little influence on the angular frequency of the high-frequency inertial modes, which are also barely modified in space (Rieutord 1995). The lower-frequency QG modes have also been considered (Vidal and Schaeffer 2015), showing that the QG modes with the largest phase velocity could penetrate into the stratified layer (in agreement with prior theory devised for rapidly rotating penetrative convection, see in Takehiro and Lister 2001; Takehiro 2015), but would have their largest amplitude in the equatorial region (in agreement with Fig. 4). Consequently, both the highest-frequency inertial modes and some large-scale QG modes (e.g., with periods of a few months) could penetrate into the stratified layer, and so may be a priori detected in geodetic or geomagnetic time-series (which are sensitive to the flow structures at the CMB).

Besides, MAC modes of the stratified layer could also generate detectable signatures in the geophysical data (Buffett 2014; Buffett et al. 2016; Buffett and Matsui 2019). However, note that the dynamics of such a layer, where double-diffusive convection or baroclinic effects (i.e., when ${\varvec{g}} \times \nabla T_0 \ne {\varvec{0}}$, e.g., due to temperature heterogeneity) could occur, remains poorly constrained and may even bring its existence on longer time scales into question.

### Observation of Magnetic Modes

Geomagnetic field variations include traveling, fluctuating, growing and decaying field features at the CMB, with a wide array of spatial wavelengths and timescales spanning a few years to a few millennia. However, it is difficult to unambiguously distinguish the part of these geomagnetic fluctuations connected to free modes versus that connected to the underlying convective dynamics. In other words, which part of the secular variation is due to free modes instead of forced motion.

A case in point is the detection of the TM. The rigid axisymmetric zonal motion involved in these modes carry axial momentum and, through coupling with the mantle, result in LOD changes. TM can then be observed jointly by inverting the rigid zonal flow variations consistent with the observed secular variation and through the observed changes in LOD. In the original study of Braginsky (Braginsky 1970), it was assumed that the period of the fundamental mode was of the order of 60-80 yr, the leading order period of the change in LOD observed over the past century, e.g., Roberts et al. (2007). This implied a $B_s$ magnetic field strength inside the core close to 0.2 mT, broadly consistent with the strength of the field at the CMB. In support of this view, not only are the multi-decadal zonal flows consistent with the observed LOD changes, e.g., Jault et al. (1988), Jackson et al. (1993), Hide et al. (2000); Pais and Hulot (2000); their spatio-temporal variations can be fit by a combination of only a few modes consistent with Equation (6a) (Zatman and Bloxham (1997), Bloxham et al. (2002), Amit and Olson (2006), Buffett et al. (2009)). However, numerical models of the geodynamo suggest that the magnetic field strength inside the core should be approximately a factor 10 larger than that at the CMB, e.g., Christensen and Aubert (2006), Schaeffer et al. (2017). Furthermore, electromagnetic coupling with the mantle is expected to attenuate TM in a few decades, e.g., Dumberry and Mound (2008), raising a concern as to whether the multi-decadal zonal flows actually represent free modes. The study of Gillet et al. Gillet et al. (2010a) showed that interannual zonal flows with a period of $\sim 6$ yr can explain a LOD signal at the same period and it is these interannual zonal flows, rather than the multi-decadal, that are now believed to represent TM. These shorter timescale TM are often referred-to as ’fast TM’ and imply an internal core field of 2 to 3 mT, more in line with our expectation based on geodynamo models. The multi-decadal zonal flows are then either forced motions by the convective dynamics, e.g., More and Dumberry (2018), or MAC waves in a stratified layer at the top of the core Buffett (2014), Buffett et al. (2016) (see Sect. 3.3). Here, it is important to note that dynamo action can also lead to *steady* zonal flows via thermal winds, as demonstrated by Aubert (2005).

Attempts at detection of non-axisymmetric core modes have been generally focused on a series of intense maxima and minima of the radial magnetic field located at low latitudes under the Atlantic hemisphere, e.g., Jackson (2003), Finlay and Jackson (2003), Finlay et al. (2010). Their typical azimuthal wavenumber is $m\approx 6$ and they are drifting westward at a rate of $\sim 17$ km/yr which has remained fairly constant over the past 400 years. These westward drifting field features may be slow MC-Rossby modes, as first proposed by Hide (Hide 1966). These are a class of MCM involving quasi-geostrophic flow columns, and a westward phase velocity as a result of the sloping boundary of the core, e.g., Jault and Finlay (2015), Bardsley (2018). Such westward drifting waves do indeed emerge in numerical models of the dynamo (Hori et al. (2015), Hori et al. (2018)). However, dynamo models typically also comprise a mean westward flow near the equator. The Coupled Earth dynamo model (Aubert et al. (2013), Pichon et al. (2016)), for instance, produces a westward drift flow as a consequence of the gravitational and electromagnetic torques acting on the inner core and mantle. This model explains the longitudinal localization of the magnetic flux patches as the result of an asymmetric buoyancy release from the inner core. Furthermore, in reconstructions of core flows, these drifting field patches can be attributed also to a westward planetary gyre that travels at low latitude in the Atlantic hemisphere, e.g., Pais and Jault (2008). If these equatorial field features are the manifestation of magnetic modes, they may then be quasi-stationary modes with very slow azimuthal phase velocities that are advected westward by the mean azimuthal flow.

The inherent difficulty in identifying magnetic modes in the secular variation is that their wave properties depend on the underlying structure of the magnetic field in the core, which is not known. An added difficulty is that crustal magnetization prevents observations of the magnetic field and its secular variation at the CMB at spatial scales smaller than spherical harmonic degree 14. These challenges imply that it may not be possible to connect unambiguously some of the observed decadal to centennial field changes with magnetic modes.

Sub-decadal low-latitude magnetic secular accelerations (or pulses) that are observed in high-resolution field models derived from satellite observations in the past few decades (e.g., Chulliat et al. (2010), Chulliat and Maus (2014), Chulliat et al. (2015)) are perhaps a clearer manifestation of non-axisymmetric core modes. These are field features dominated by spherical harmonic degree 5 to 6 at the CMB which appear to be oscillating as a standing wave with a period of approximately 6 yr (Chulliat et al. (2010), Chulliat and Maus (2014)), although fast eastward and westward longitudinal drift velocities cannot be ruled out (Chulliat et al. 2015). As mentioned in the previous section, these may represent equatorially trapped MAC waves in a stratified layer at the top of the core, e.g., Bergman (1993), Knezek and Buffett (2018), Buffett and Matsui (2019). However, non-axisymmetric oscillating quasi-geostrophic flow structures can explain these pulses (Kloss and Finlay 2019) and these may instead represent subdecadal quasi-geostrophic MC modes (Gerick et al. 2021), which do not require stratification, or QG Alfvén waves (Aubert and Finlay 2019), travelling along a strongly heterogeneous magnetic field near buoyant plumes. Whether MAC waves, QG MC modes or QG Alfvén waves, these oscillating pulses, dominated by magnetic energy, offer the best prospect to connect non-axisymmetric magnetic modes to observed time-dependent magnetic field features at the CMB. For a more detailed revision on the fast inter-annual secular variations and the possible role of modes in the core, we refer the reader to Chapter (XXX Gillet et al.) of this series.

### Coupling at the CMB and Impact on Earth’s Rotation

#### Changes in Earth Orientation

The source of the damping of the Earth’s rotational modes has not been established with certainty. One reason is that a comprehensive model is still beyond reach, and a meaningful comparison with observation is yet to be achieved. The damping of the FCN, for instance, can be affected by viscous and Ohmic dissipation (Buffett 1992), the latter taking place both in the fluid core and in a conductive layer at the bottom of the mantle. The small scale topography of the CMB definitely plays a role, but it is not yet known to what extent. These effects are, or course, in addition to the energy dissipated within the inner core or the mantle.

The inferred FCN damping factor, deduced from the retrograde annual nutation (Koot et al. 2010), cannot be explained unambiguously yet. One of the difficulties is that we do not have a reliable estimate of the non-dipolar radial magnetic field at the CMB. Downward continuation of the observed magnetic field at the Earth’s surface is limited to a maximum spherical harmonic degree $l_\mathrm {max}=13$ due to the magnetization of the Earth’s crust. The radial part of the dipolar field at the CMB is known, $B_r^\mathrm {D}=0.21$ mT (rms), but the radial non-dipolar component $B_r^\mathrm {ND}$ is not well constrained. An estimate of $B_r^\mathrm {ND}$ at the CMB is obtained by assuming the same spectral trend of the magnetic field as observed at the surface, which gives $B_r^\mathrm {ND}=0.28$ mT (rms). To explain the damping of the FCN solely in terms of electromagnetic coupling requires $B_r^\mathrm {ND}\gtrsim 0.63$ (rms) and a 200-m-thick conductive layer at the bottom of the mantle with an electrical conductivity similar to that of the fluid core (Koot et al. (2010), Buffett (1992), Buffett et al. (2002)). Thus, either the magnetic field at the CMB has considerable more energy in the small scales than what is expected from downward continuation, or there is another dissipative mechanism affecting the FCN, for instance, viscous dissipation. Note that the magnetic field has relatively tight amplitude constraints deriving from power availability considerations (Christensen and Aubert (2006), Buffett and Christensen (2007)). If electromagnetic coupling is weak, viscous dissipation in the boundary flow can account by itself for the observed FCN damping provided the effective Ekman number is near $E\sim 10^{-11}$, which is four orders of magnitude larger than that based on molecular viscosity ($E\sim 10^{-15}$) (Deleplace and Cardin (2006), Mathews and Guo (2005), Palmer and Smylie (2005)). An enhanced eddy viscosity has been invoked (e.g., Deleplace and Cardin (2006), Palmer and Smylie (2005)) to explain the required Ekman number, but the time and length scales of the turbulent eddies are not consistent with the flow associated with the FCN (Buffett and Christensen (2007)).

Compounding the puzzle, Earth’s precession may induce boundary layer turbulence at the CMB. This is because the spin axis of the precessing fluid core is slightly misaligned from that of the mantle. As seen from the rotating mantle frame, the core is precessing with a period of one day, leading to a diurnal differential motion at the CMB of the order of 4 mm/s (Tilgner 2015; Pais and Le Mouël 2001). The Reynolds number associated with this differential motion ($Re\simeq 473$) (Tilgner (2015), Le Bars et al. (2015)) could be sufficiently high to induce turbulence (Sous et al. (2013), Cébron et al. (2019)) (although see also Buffett (2021)). Turbulence may be further enhanced by topographic features. Thus, even if the FCN flow by itself may not induce turbulence, the turbulent eddies generated by the differential core–mantle precession may affect the viscous dissipation of the FCN. This is a challenging question well worth exploring.

Damping of the FICN is also required in order to explain Earth’s nutation observations. Electromagnetic coupling at the ICB can account for this damping, but it requires a high radial magnetic field amplitude in excess of 7 mT (Buffett et al. (2002), Koot et al. (2010), Koot and Dumberry (2013)). Viscous relaxation within the volume of the inner core can explain a part of the FICN damping, although this requires a bulk inner core viscosity smaller than $10^{15}$ (Pa s Koot and Dumberry (2011), Greff-Lefftz et al. (2000)).

Though a high ICB magnetic field or a low inner core viscosity cannot be ruled out, dissipation in the volume of the fluid core has been pursued as an alternative explanation. Core flow is induced by the flattened and wobbling inner core. The Ohmic dissipation within the fluid core, mostly taking place in internal shear layers emanating from the ICB, was investigated in the study by Buffett (Buffett 2010b). This model employs spherical boundaries, a uniform magnetic field, and a radial flow at the ICB forced with the dimensional (nominal) frequency of the FICN $\omega _\textsc {ficn}=-\varOmega _0(1-\epsilon )$, where $\epsilon =0.0025$ is the hydrostatic flattening of the inner core. Numerical results for Ekman number values as low as $E\sim 10^{-7}$ suggest an Ohmic dissipation scaling as $E^{-2/3}$, and to match the observed damping of the FICN, the rms magnetic field in the core must be approximately 2.5 mT, in good agreement with our expectation based on the propagation speed of Alfvén waves (Gillet et al. 2010b). This idea was pursued further by Lin and Ogilvie (2020) using a similar model, with a more satisfactory treatment of the Lorentz force, and obtaining solutions for Ekman numbers as low as $E\sim 10^{-11}$. They reproduced Buffett’s results for $E\gtrsim 10^{-7}$; however, they showed that at lower Ekman numbers the Ohmic dissipation deviates considerably from Buffett’s scaling. When extrapolated to Earth’s parameters, the total dissipation is too low by a few orders of magnitude to explain the observed FICN damping.

It should be noted that the back reaction of the flow on the inner core wobbling motion is not included in these models. In other words, the mode spectrum contains only *inertial* eigenmodes and not the FICN. At very low Ekman numbers, it becomes easier to excite inertial mode resonances, the lower the Ekman number the sharper and more numerous they become. Whether or not a mode is excited by an imposed forcing depends sensitively on the chosen frequency (Ogilvie 2013). Introducing a forced radial flow at the ICB is then bound to excite inertial modes in the outer core depending on the chosen forcing frequency. This will tell us more about the inertial eigenmode spectrum of the core rather than about the FICN. A true coupled model in which the inner core is able to respond to torques is a better representation of the dynamics. The FICN in such a model would be a true eigenmode of the system (along with the inertial modes), as in the coupled 2-layer model discussed in Sect. 5.

#### Changes in Length of Day

The temporal changes in the axial angular momentum of the core can be computed on the basis of zonal flows at the surface of the core, provided they extend rigidly inside the core. Comparisons between the predicted changes in LOD computed from these flows agree well with the observed changes at decadal Jault et al. (1988), Jackson et al. (1993), Hide et al. (2000), Pais and Hulot (2000) and interannual timescales Gillet et al. (2010a), Gillet et al. (2015). Not only this indicates that the zonal flows are predominantly rigid, it also demonstrates that LOD variations at both these timescales are caused by an exchange of angular momentum between the core and the mantle.

However, the nature of the torque that allows for this exchange remains a question mark. Most of the efforts have focused on the decadal torque, e.g., Jault (2003), Roberts and Aurnou (2012), but here we direct our attention to interannual periods connected with the TM. An estimate of the torque on the mantle required to produce LOD variations of 0.1 ms at a period of 6 yr is $\sim 2 \times 10^{17}$ N m, e.g., Figure 1 of Roberts and Aurnou (2012).

Electromagnetic (EM) coupling at the CMB has long been suggested as a possible mechanism of core–mantle angular momentum exchange, e.g., Rochester (1960). Gillet et al. (Gillet et al. 2017) have shown that, when using a radial field strength at the CMB and a lowermost mantle conductance consistent with those required to explain the damping of the FCN, EM coupling transforms standing TM into outward travelling Alfvén waves similar to those imaged in Earth’s core. They further noted that the strength of EM coupling is consistent with the required core–mantle torque to produce the observed LOD signal, although they did not present an actual calculation.

EM coupling at the ICB is expected to be strong, e.g., Gubbins (1981), and TM should induce fluctuations in inner core rotation. In fact, the whole of the cylinder that encloses the inner core (the tangent cylinder) is expected to rotate almost as a rigid body, e.g., Buffett (1998). A longitudinal rotation of the non-axisymmetric shape of the inner core by an angle $\alpha$ exerts a gravitational torque on the mantle equal to $\varGamma \alpha$, where $\varGamma$ is a constant that depends on the mass anomalies in the mantle, e.g., Buffett (1996). The latest estimate of $\varGamma$ ranges from $3 \times 10^{19}$ to $2 \times 10^{20}$ N m Davies et al. (2014). A mean zonal flow ${\bar{u}}_\phi$ inside the tangent cylinder (radius $r_s$) oscillating at frequency $\omega$ is connected to $\alpha$ by ${{{\bar{u}}}}_\phi = r_s \omega \alpha$. Taking ${{{\bar{u}}}}_\phi =0.4$ km/yr as a typical measure for zonal flows with a period of 6 yr, see figure 11 of Gillet et al. (2019) gives $\alpha \approx 3 \times 10^{-4}$ rad or 0.018$^\circ$, which gives a gravitational torque amplitude between $9\times 10^{15}$ to $6\times 10^{16}$ N m. This is slightly below, but sufficiently close to the required torque to suggest that it may play a role. This is provided the inner core topography does not relax viscously on a timescale shorter than 6 yr. On a related note, the MICG mode (see Sect. 4) has been suggested as a possible explanation for the 6 yr LOD (Mound and Buffett 2006). However, the successful prediction of the 6-yr LOD reconstructed from core flows by Gillet et al. (2010a) shows that the angular momentum exchange is between the fluid core and the mantle, not between the inner core (in fact the whole tangent cylinder) and the mantle as it would be the case for a pure MICG mode. Hence, although the MICG mode may help to amplify the oscillation, its coupling with the fluid outside the tangent cylinder cannot be ignored.

The topography of the CMB likely features peaks and troughs of the order of a few km (Koelemeijer 2020) and longitudinal pressure variations associated with core flows acting on these induce a topographic torque on the mantle, e.g., Hide (1969). The latest effort to model this torque is the study of Gerick et al. (2020) who developed a QG model in an ellipsoidal core, similar to that which has been used in 3-D hydromagnetic models (Vidal et al. 2019, 2020). They show that the longitudinal pressure variation associated with the TM scales as $p\sim \rho {{{\bar{u}}}}_\phi v_A$, where $v_A$ is the mean cylindrical radial Alfvén velocity in the core and ${{{\bar{u}}}}_\phi$ the azimuthal velocity of the TM. Taking ${{{\bar{u}}}}_\phi \approx 5\times 10^{-6}$ m $\hbox {s}^{-1}$ and $v_A \approx 2\times 10^{-2}$ m $\hbox {s}^{-1}$ (Gillet et al. 2010b, 2015), gives $p \approx 10^{-3}$ Pa. Acting on the equatorial ellipticity of the CMB, this gives a pressure torque of the order of $10^{14}$ N m. Considering smaller longitudinal wavelength may increase the amplitude of this torque, but it is unclear whether it can reach the required target of $2\times 10^{17}$ N m.

An alternate form of pressure torque might result from the combined effects of stratification, magnetic field and topography. Pockets of strongly stratified core fluid may be trapped by undulations of the CMB, largely inhibiting the flows in such pockets. TM would exert a strong EM traction on these pockets, which would then exert a pressure force on their enclosing topography (Glane and Buffett 2018). While this mechanism remains to be confirmed with more complete models (Jault 2020), this would mimic EM coupling with a highly electrically conducting lower mantle.

### Nonlinear Couplings of Modes

Beyond the possible direct observations of modes, one can wonder whether the fluid modes could play a role in the long-term dynamics of the outer core (which could have measurable effects in the observations of the Earth’s rotation or geomagnetic data). Indeed, when two free modes of angular frequencies $\omega _{1,2}$ are excited on long-enough time scales, they can often be coupled with a primary oscillating flow ${\varvec{U}}_0 \propto \exp (\mathrm {i} \omega _0 t)$, through the nonlinear operator $({\varvec{v}}\varvec{\cdot } \nabla ) {\varvec{v}}$ of the momentum equation. Such mode couplings are known to occur when appropriate resonance conditions are satisfied, that can be obtained by linearizing Equations (1) around ${\varvec{U}}_0$. This yields, for instance, in the frequency domain (see the mathematical derivation in Tilgner 2015)

14 $$\begin{aligned} \omega _1 \pm \omega _2 = \omega _0. \end{aligned}$$

Note that diffusive effects can allow less stringent temporal resonant conditions to be fulfilled (see a discussion of these effects in Kerswell 2002 which are important for numerical simulations or experiments). Spatial resonance conditions also exist, but they can only be written explicitly in some simple geometries (e.g., $m_1 \pm m_2 = m_0$ in axially symmetric geometries). When resonance conditions are satisfied, usually flow instabilities can grow on longer time scales (when the instability growth rate is larger than the diffusive damping rates), and then higher-order nonlinear terms will come into play to obtain a saturated (possibly turbulent) state. Such resonant couplings could play an important dynamical role in planetary cores, leading to space-filling turbulence (e.g., Le Reun et al. 2017; Grannan et al. 2017; Lemasquerier et al. 2017), generating geostrophic flows (e.g., Kerswell 1999; Brunet et al. 2020; Le Reun et al. 2019, 2020), enhancing dissipation (e.g., Cébron et al. 2019; Vidal et al. 2019), or even sustaining magnetic field generation through dynamo action (Cébron and Hollerbach 2014; Reddy et al. 2018; Vidal et al. 2018). To explore quantitatively such dynamical scenarios for the core, an accurate description of the core modes (including their damping rates) is required, which is still beyond the state-of-the-art models that have been developed so far. Thus, many uncertainties remain in extrapolating the previous works for geophysical applications. We review below a few open questions.

The inertial modes are ideal candidates to satisfy resonant conditions (14), since they play a central role in the theory of rapidly rotating fluids (Greenspan 1968; Zhang and Liao 2017). The outer core is indeed known to be rapidly rotating as measured by the smallness of the Rossby number ${{{\mathcal {R}}}}o = U/(\varOmega _0 R_o) \sim 10^{-6} - 10^{-5}$ with *U* the typical amplitude of core flows at the CMB (Holme 2015), which compares inertia and the Coriolis force in the momentum equation. Moreover, turbulent convection is not expected to strongly enhance the diffusive damping of the inertial modes (e.g., in other contexts Wu 2005; Ogilvie and Lin 2007), because of the huge separation of time scales (see Fig. 3) between the short periods of the inertial modes and the turnover time scale of the convective eddies (see the discussion in Tilgner 2015).

Several primary flows ${\varvec{U}}_0$ could then be invoked to satisfy resonant conditions (14). Orbital forcings (e.g., tides, precession or librations) can notably drive suitable large-scale oscillating flows in non-spherical planetary cores (e.g., Le Bars et al. 2015; Tilgner 2015), which typically vary on diurnal time scales in the inertial frame (see Fig. 3). Since convection varies on much longer time scales in the core (typically a few decades or even longer), turbulent convection is not expected to be efficient in damping these large-scale orbitally driven flows (Goodman and Oh 1997; Vidal and Barker 2020). Hence, these orbital flows with nearly diurnal frequencies certainly coexist with turbulent convection on very long time scales, and several pairs of inertial modes could be involved in (14) to trigger flow instabilities. Different scenarios have thus been explored in neutrally buoyant fluids, for instance for tides (Grannan et al. 2017; Vidal and Cébron 2017; Vidal et al. 2019), librations, e.g., Vantieghem et al. (2015), Lemasquerier et al. (2017), or precession (Kerswell 1993b; Tilgner 2015; Lin et al. 2015; Cébron et al. 2019).

However, considerable work remains to be done to rigorously extrapolate these previous results for the outer core. For instance, little work has been done to incorporate background turbulence (e.g., Fabijonas and Holm 2003, in unbounded geometries). Preliminary numerical (Cébron et al. 2010; Wei and Tilgner 2013) and experimental (Lavorel and Le Bars 2010) studies have, however, reported that such instabilities may grow upon weakly turbulent convection, but their findings remain to be confirmed (notably in the presence of fully turbulent rotating convection). The amplitude of precession and tidal forcings in the core may also be too weak to overcome the viscous and Ohmic diffusion in the core (Kerswell 1994), which would thus inhibit the flow instabilities. In this regard, these mechanisms should be urgently explored in shell geometries (as in Lemasquerier et al. 2017), since the damping of the inertial modes strongly differs in the presence of an inner core. Note, however, that the situation may have been more favorable in the Early Earth, when the Moon was orbiting closer (e.g., Cébron et al. 2012b).

In addition, resonant couplings may also occur due to the interactions of two inertial modes with another primary inertial mode ${\varvec{U}}_0$ (known as triadic interactions), for instance, driven by core convection. Although the typical turnover frequency of the convective eddies is believed to be much smaller than the frequency of the inertial modes, turbulent rotating convection might also sustain higher-frequency flows compatible with inertial waves (Lin 2021). This scenario is still very speculative and deserves further work. Nonetheless, these preliminary results may pave the way for future progress in the physical understanding of core convection.

To conclude our overview of nonlinear mode couplings, it is worth mentioning the possible role of the other fluid modes. Resonant couplings between one inertial mode, one slow MC modes and the tidally driven flow have also been considered, but it has been shown that Ohmic diffusion is too large compared to the forcing amplitude for such couplings to sustain any flow instabilities for realistic planetary core conditions (Kerswell 1993a; Vidal et al. 2019). Resonant couplings of only slow MC (or Alfvén) modes with orbitally driven flows cannot satisfy conditions (14), because their typical frequency (see Fig. 3) is much smaller than the nearly diurnal frequency $\omega _0$ (in dimensional units). Yet, nonlinear interactions of these slow modes with convection should be explored (because convection can excite smaller frequencies $\omega _0$ than for orbital forcings). Besides resonant couplings, nonlinear self-interactions of slow magnetic modes might also occur in the core (Hori et al. 2020), yielding flow structures that might be broadly consistent with some core flows inferred from geomagnetic data. In stably stratified fluids, resonant couplings between inertial-gravity waves and tidal flows are also expected (Vidal et al. 2019). They are not directly relevant for the core that currently undergo convection, but such resonant interactions may have occurred in the Early Earth if it were stably stratified in density (Nimmo 2015; Jacobson et al. 2017).

## Conclusions and Future Outlook

We have presented some of the most salient aspects of the Earth’s eigenmodes encompassing its fluid core and its rotational variations from diurnal to inter-annual time scales. Different observables such as length-of-day variations, nutations, magnetic field variations are intricately related to the underlying structure of the core and the lowermost layer of the mantle. Although specialized models have been developed to tackle some of these specific problems, as we showcase in this review, major challenges still remain in the way to obtain a synergistic view. We discuss some of these challenges and perspectives in the following paragraphs.

Since compressibility effects are often assumed to be small in the Earth’s core, the fluid modes of the outer core are usually modeled by using the Boussinesq approximation. The simplicity of this approximation is a real numerical advantage to compute the 3-D modes of the core, but the validity of the Boussinesq approximation is questionable for the Earth’s core (Anufriev et al. 2005). Recent fully compressible eigenmodes computations have, for instance, shown that compressibility in the core may significantly affect the frequencies of some inertial modes (Seyed-Mahmoud et al. 2007, 2015; Vidal and Cébron 2020). The density variations may also favor the couplings between the rotational modes of the Earth and the inertial modes (Toomre 1974). In the next generation of core oscillations models, more accurate sound-proof approximations could thus be used (e.g., the anelastic approximation (Anufriev et al. 2005)). Such approximations would allow a more accurate description of the outer core (to obtain more accurate predictions for geophysical applications) and would be computationally less demanding to solve than the fully compressible model.

The next generation of models should further consider non-spherical boundaries. Much effort has been recently devoted to explore the effects of the large-scale ellipsoidal deformation, but smaller-scale wavelengths would be also worth modeling. Non-standard numerical strategies ought to be designed to this end, which is still an active field of research.

Many questions remain on the coupling between the core and mantle. This is important, for instance, to understand better the nature of the torque involved in decadal and sub-decadal LOD changes. But core–mantle coupling ultimately plays a role, even if minor, for all the core modes that we have discussed in this review. As mentioned in the previous paragraph, the effect of topographic coupling on core modes remains largely unexplored. Electromagnetic coupling at the CMB may be responsible for the damping of the FCN (Buffett (1992), Buffett et al. (2002), Koot and Dumberry (2013)), can account for a part of the LOD changes (Rochester (1960), Holme (1998a), Holme (1998b)) and can explain some attributes of the observed Alfvén waves in the core (Schaeffer and Jault (2016), Gillet et al. (2017)) and perhaps even a part of the large-scale core flow geometry (Dumberry and More (2020)). Collectively, these studies suggest a conductance of $1-3 \times 10^8$ S at the base of the mantle, but the diurnal timescale of the nutations requires the electrical conductivity to be close to that of the core ($10^5 - 10^6$ S/m) and concentrated in a thin layer of a few hundred meters. Several ideas for how the lowermost mantle may be enriched in iron have been proposed (Petford et al. (2005), Kanda and Stevenson (2006), Otsuka and Karato (2012), Dobson and Brodholt (2005), Labrosse et al. (2007)), but it remains unclear whether a conductivity approaching that of the core is possible. An alternative explanation, that pockets of strongly stratified core fluid trapped by undulations of the CMB may mimic a high lowermost mantle conductivity Glane and Buffett (2018), Jault (2020), is attractive in this context, but this requires further testing.

It is intriguing that the LOD variations associated with core zonal flows inferred from geomagnetic data are less correlated with the observations in the recent satellite era than in previous decades (Gillet et al. 2019). This might be connected to the inference of flows in high-latitude regions, where geomagnetic data are more polluted by ionospheric currents and the presence of an inner core cannot be neglected. The propagation of torsional Alfvén waves through the tangent cylinder or the coupling between these two regions is crucial in understanding these discrepancies. The significance of topographic coupling for torsional Alfvén modes remains open despite recent insights suggesting that it is inefficient in a full ellipsoidal core (Gerick et al. 2020). This conclusion could change in the presence of an inner core. Whether electromagnetic and topographic coupling of the torsional Alfvén waves/modes with an inner core is significant needs to be further investigated. Another possibility of increased topographic coupling for torsional Alfvén modes is, as mentioned before, smaller scale topography of the CMB or the presence of non-closed geostrophic contours, so that angular momentum is carried by Rossby waves instead of the geostrophic flow.

The observation of Earth’s rotational variations, continuously improving over the years, is offering us a window to processes taking place in the core. Thus, it is key to incorporate the right physics into current models to take full advantage of this opportunity. We have seen, for instance, that eigenmodes can influence each other when their frequencies are close, affecting even their damping. This might be particularly relevant in the busy diurnal frequency band where some rotational and inertial modes must coexist along with external (tidal) forces. A proper understanding of this mechanism will shed light into the nature of the coupling between the fluid core and the mantle. We still lack a clear picture of the physical characteristics at either side of the CMB. Is there an electrically conductive layer at the bottom of the mantle? A stratified layer at the top of the core? What is the roughness of the CMB, and does it lead to turbulence? How much power is dissipated? Models addressing these issues will also improve our understanding of longer time-scale processes such as the length-of-day variations and the geomagnetic jerks. A true interdisciplinary approach would be a great tool to confront these challenges.
